# The neurosteroid pregnenolone is synthesized by a mitochondrial P450 enzyme other than CYP11A1 in human glial cells

**DOI:** 10.1016/j.jbc.2022.102110

**Published:** 2022-06-07

**Authors:** Yiqi Christina Lin, Garett Cheung, Edith Porter, Vassilios Papadopoulos

**Affiliations:** 1 Department of Pharmacology and Pharmaceutical Sciences, School of Pharmacy, University of Southern California, Los Angeles, California, USA; 2 Department of Biological Sciences, California State University, Los Angeles, California, USA

**Keywords:** steroidogenesis, human glial cells, pregnenolone, CYP11A1, neurosteroid, reactive oxygen species, cytochrome P450, 20α-HC, 20α-hydroxycholesterol, 22(R)-HC, 22(R)-hydroxycholesterol, 22(S)-HC, 22(S)-hydroxycholesterol, AMG, aminoglutethimide, CNS, central nervous system, CYP, cytochrome P450, DHEA, dehydroepiandrosterone, FBS, fetal bovine serum, IS, internal standard, ISH, *in situ* hybridization, KC, ketoconazole, MS, mass spectrometry, NHA, normal human astrocyte, PBST, PBS with Tween-20, qRT-PCR, quantitative RT-PCR, RIPA, radioimmunoprecipitation assay, ROS, reactive oxygen species, SNAP, S-nitroso-N-acetylpenicillamine, Trolox, (±)-6-hydroxy-2,5,7,8-tetramethylchromane-2-carboxylic acid

## Abstract

Neurosteroids, modulators of neuronal and glial cell functions, are synthesized in the nervous system from cholesterol. In peripheral steroidogenic tissues, cholesterol is converted to the major steroid precursor pregnenolone by the CYP11A1 enzyme. Although pregnenolone is one of the most abundant neurosteroids in the brain, expression of CYP11A1 is difficult to detect. We found that human glial cells produced pregnenolone, detectable by mass spectrometry and ELISA, despite the absence of observable immunoreactive CYP11A1 protein. Unlike testicular and adrenal cortical cells, pregnenolone production in glial cells was not inhibited by CYP11A1 inhibitors DL-aminoglutethimide and ketoconazole. Furthermore, addition of hydroxycholesterols increased pregnenolone synthesis, suggesting desmolase activity that was not blocked by DL-aminoglutethimide or ketoconazole. We explored three different possibilities for an alternative pathway for glial cell pregnenolone synthesis: (1) regulation by reactive oxygen species, (2) metabolism *via* a different CYP11A1 isoform, and (3) metabolism *via* another CYP450 enzyme. First, we found oxidants and antioxidants had no significant effects on pregnenolone synthesis, suggesting it is not regulated by reactive oxygen species. Second, overexpression of CYP11A1 isoform b did not alter synthesis, indicating use of another CYP11A1 isoform is unlikely. Finally, we show nitric oxide and iron chelators deferoxamine and deferiprone significantly inhibited pregnenolone production, indicating involvement of another CYP450 enzyme. Ultimately, knockdown of endoplasmic reticulum cofactor NADPH-cytochrome P450 reductase had no effect, while knockdown of mitochondrial CYP450 cofactor ferredoxin reductase inhibited pregnenolone production. These data suggest that pregnenolone is synthesized by a mitochondrial cytochrome P450 enzyme other than CYP11A1 in human glial cells.

The term neurosteroids refer to steroid hormones synthesized in the central or peripheral nervous system, either *de novo* from cholesterol or metabolically *in situ* from precursors in the blood (1). These neurosteroids—such as pregnenolone, dehydroepiandrosterone (DHEA), and their sulfates—can accumulate in the nervous system independently of peripheral gland secretion and have important roles in modulating neuronal functions and behavior. Changes in neurosteroid levels have been implicated in neurological and psychiatric disorders, such as Alzheimer’s disease and mood disorders, respectively (2). Levels of pregnenolone and DHEA can be elevated in brains of Alzheimer’s disease patients compared to cognitively intact control subjects (3, 4), while levels of the downstream neurosteroids allopregnanolone, pregnenolone sulfate, and DHEA sulfate were found to be lower in brains of Alzheimer’s disease patients (3, 4, 5). In mood disorders such as depression and bipolar disorder, neurosteroid levels in the brain have generally been found to be reduced, and this effect can be reversed with antidepressant or lithium treatment (6). With the approval of brexanolone, an allopregnanolone-based drug, for treatment of postpartum depression (7), there is increasing interest in pharmacological development of neurosteroids to treat neurological disorders.

In the brain, neurosteroids exert their effects not only through classical genomic mechanisms but also through modulation of neurotransmitter receptors, such as GABA_A_ and glutamate receptors. For example, allopregnanolone is a potent positive allosteric modulator of GABAergic neurotransmission and was found to have anxiolytic effects (8, 9). Neurosteroids such as DHEA and pregnenolone sulfate can modulate activity of N-methyl-D-aspartate receptors and have been proposed to play important roles in neuronal plasticity and neuronal cell differentiation (10, 11, 12). Pregnenolone not only serves as the precursor to all other neurosteroids but also has physiological and pharmacological effects on its own (for review, see (13)). For example, administration of pregnenolone can enhance memory in mice (14, 15). In humans, administration of pregnenolone was shown to improve symptoms in patients with bipolar disorder and schizophrenia (16, 17), suggesting that this steroid has a neuromodulatory role in the brain. Pregnenolone may also be involved in modulating neuronal shape and plasticity by stimulating microtubule assembly (18, 19). Furthermore, pregnenolone can protect neurons from glutamate-induced and amyloid beta–induced toxicity (20). In general, neurosteroids are believed to reduce neuroinflammation and be neuroprotective (21, 22). The overall effects of neurosteroids on the brain depend on the extent that they are metabolized and how the parent steroid and its metabolite(s) affect intracellular or extracellular receptors (2). Therefore, understanding the metabolic pathway of neurosteroids and how the major neurosteroid precursor pregnenolone is synthesized should allow better understanding of how neurosteroid-modulating drugs may affect brain function.

Steroid synthesis involves activity of multiple enzymes, most of which are highly expressed in the adrenals and gonads but have also been shown to be present in the brain (23, 24). In the classical steroidogenic pathway, *de novo* synthesis of steroids requires conversion of cholesterol to pregnenolone by the cytochrome P450 (CYP) 11A1 enzyme (CYP11A1). Cholesterol is first transported into the mitochondria, mediated by translocator protein 18 kDa (TSPO) and steroid acute regulatory protein (STAR) (25). In the inner mitochondrial membrane, CYP11A1 performs two successive hydroxylations on C22 and C20 of cholesterol, forming the intermediates 22(R)-hydroxycholesterol (22(R)-HC) and 20α,22(R)-dihydroxycholesterol, followed by cleavage of the cholesterol side chain between C20 and C22 to form pregnenolone (26) (Fig. 1*A*). This enzyme activity requires one molecule of oxygen and one molecule of NADPH for each reaction. Unlike CYP450 enzymes in the endoplasmic reticulum that use NADPH-CYP reductase (POR) as a redox partner, mitochondrial CYP11A1 activity involves two cofactors ferredoxin (FDX1) and ferredoxin reductase (FDXR), also known as adrenodoxin and adrenodoxin reductase, respectively, similar to other mitochondrial CYP450s (27). CYP11A1 uses electrons from the electron transport chain to carry out reactions, delivered through NADPH (28). The electron on NADPH is transferred to ferredoxin reductase, which subsequently donates it to ferredoxin. Ferredoxin then diffuses into the mitochondrial matrix to shuttle the electron to the heme group on CYP11A1, allowing the enzyme to carry out its side chain cleavage activity.

**Figure 1:**
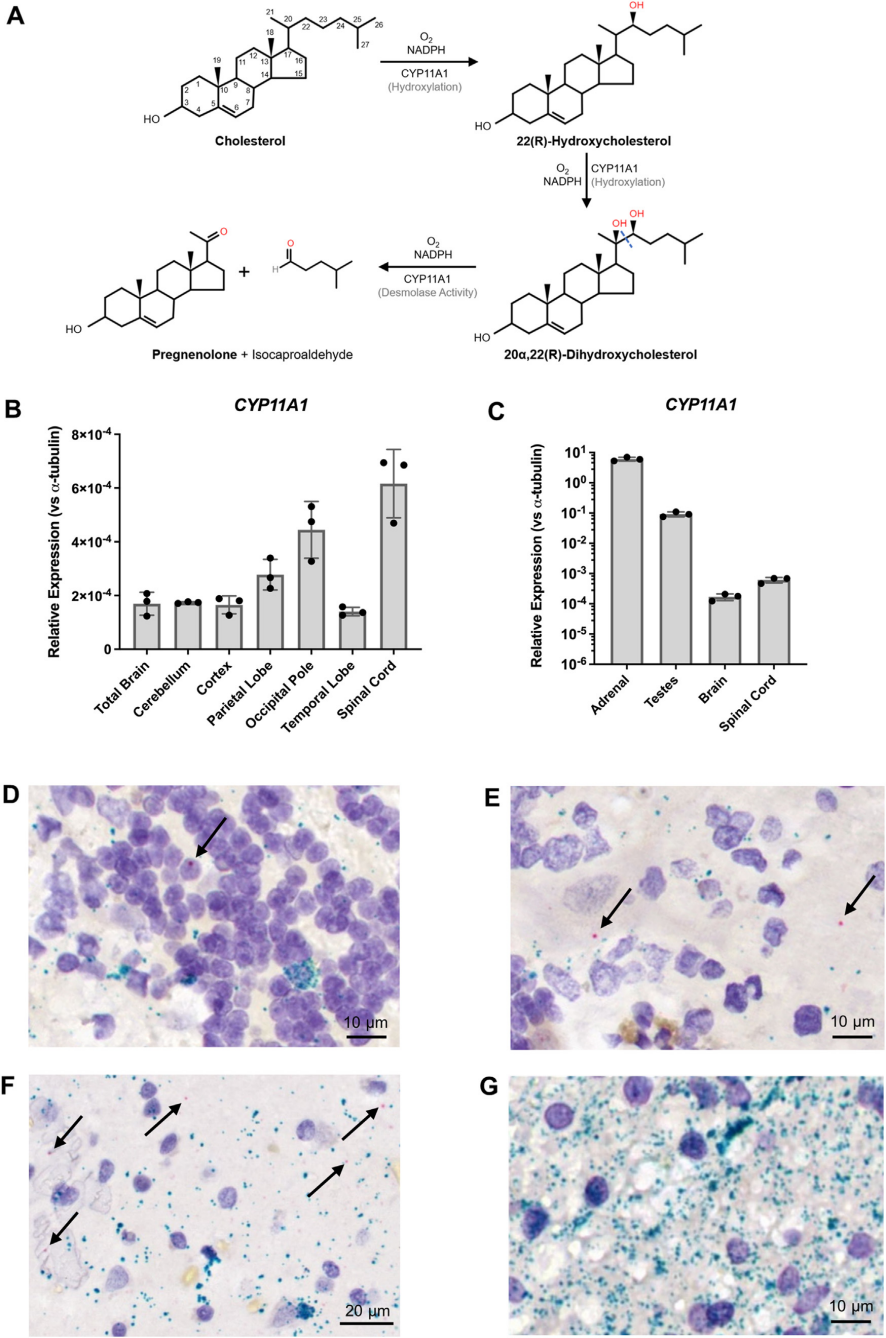
**mRNA expression of *CYP11A1* in human brain tissues**. *A*, schematic diagram describing the cholesterol side-chain cleavage reaction by CYP11A1. qRT-PCR analyses of *CYP11A1* in (*B*) total brain, cerebellum, cortex, parietal lobe, occipital pole, temporal lobe, and spinal cord and (*C*) human testes, adrenal glands, brain, and spinal cord. Data for brain and spinal cord in (*C*) are the same as the total brain and spinal cord data in (*B*), respectively. Gene expression is shown as relative expression to α-tubulin. Data are presented as mean ± SD, N = 3. Each data point represents a replicate of the same RNA sample. (*D*–*G*) RNAscope *in situ* hybridization analyses of human cerebellum and cerebral cortex tissue (male, 63-years-old). A chromogenic assay was performed with two targets: CYP11A1 (*red*) and myelin basic protein (MBP; *teal*). Each punctate dot represents one molecule of mRNA. CYP11A1-positive cells can be found in the granule layer (*D*), molecular layer and Purkinje layer (*E*) of the cerebellum, as well as gray matter of the cortex (*F*), indicated by *black arrows*. No CYP11A1-positive cells were observed in white matter (*G*). Little colocalization between MBP and CYP11A1 can be observed. qRT-PCR, quantitative RT-PCR.

Even though pregnenolone is the most abundant steroid in the brain (5), CYP11A1 expression has been difficult to detect, particularly in the human brain. Studies have reported the presence of *Cyp11a1* mRNA in rat brains at approximately 0.01% of adrenal *Cyp11a1* mRNA levels (29). CYP11A1 appears to be more highly expressed in oligodendrocytes in the rat brain compared to astrocytes and neurons, which is supported by higher pregnenolone production in oligodendrocytes (30). This led to the hypothesis that oligodendrocytes produce the majority of steroid precursors, which are metabolized to other neurosteroids by astrocytes and neurons. In human brains, *CYP11A1* mRNA was reported to be present in the particular brain regions such as the hippocampus but levels range from 200 to 10,000 times lower than that of the adrenals (31, 32). Despite multiple reports of *CYP11A1* mRNA presence in the human brain and of Cyp11a1 protein in rat brains, protein expression of this enzyme in the human brain has been inconclusive (see (33) for review). To our knowledge, there have only been two reports of CYP11A1 protein in the human brain, but the presence was only demonstrated in small areas of the cerebellum and frontal cortex in a few numbers of cells (34, 35). The levels of pregnenolone found in the brain and the ability for the brain to produce increased amounts of pregnenolone in response to treatment with TSPO ligands (13, 36) creates a discrepancy with the local CYP11A1 levels, suggesting that alternative pathways may be used to produce pregnenolone in the brain (33). To examine this discrepancy, we determined the expression of *CYP11A1* in four human glial cell lines. We found that CYP11A1 protein can only be detected by immunocytochemistry with very weak signals and, thus, investigated whether a CYP11A1-independent pathway is used by brain cells to produce pregnenolone.

## Results

### Evaluating the steroidogenic potential of glial cells

Previous studies suggested that oligodendrocytes produce more pregnenolone than astrocytes and neurons (30, 37); thus, we used the human glioma cell lines MGM-1 and MGM-3 as the main models for this study. We also used the human astrocyte cell line normal human astrocyte (NHA) and human microglia cell line HMC3 as noncancerous glial cell models to confirm our findings. For comparison to peripheral steroidogenic cells, we used the human adrenal cortical carcinoma cell line H295R-S1 and mouse Leydig tumor cell line MA-10. The characteristics of each cell line used are summarized in Table S1.

To determine whether human glial cells express the components of the machinery used for peripheral steroid biosynthesis, we performed quantitative RT-PCR (qRT-PCR) to evaluate expression of genes important for steroidogenesis. In general, enzymes in the classical steroidogenesis pathway (Fig. S1*A*) are expressed at very low levels in glial cells (Figs. 1*B* and S1*B*). Enzymes further upstream in the steroidogenesis pathway are more highly expressed. Glial cells express low but detectable amounts of mRNA for *CYP11A1*, *CYP17A1*, *CYP19A1*, 3β-hydroxysteroid dehydrogenases (*HSD3B1*, *HSD3B2*), 17β-hydroxysteroid dehydrogenases (*HSD17B1*, *HSD17B2*, *HSD17B3*, *HSD17B4*, *AKR1C3*), 3α-hydroxysteroid dehydrogenase (*AKR1C1*, *AKR1C2)*, 5α-reductase (*SRD5A1*, *SRD5A2*), steroid sulfatase (*STS*), and sulfotransferase (*SULT2B1*). No expression for *CYP11B1*, *CYP11B2*, *CYP21A2*, *AKR1C4*, and *SULT2A1* could be detected in glial cells.

### Evaluating *CYP11A1* mRNA expression in the human brain

We determined the overall levels of *CYP11A1* mRNA in various regions of the human central nervous system (CNS) using qRT-PCR. The highest expression of *CYP11A1* was found in the spinal cord, followed by the occipital pole and the parietal lobe (Fig. 1*B*). The expression in the temporal lobe, cerebral cortex, cerebellum, and total brain were approximately the same. *CYP11A1* expression in CNS tissue is more than 1000 times lower than in the adrenals and more than 100 times lower than in the testes (Fig. 1*C*).

To further investigate the tissue and cell type localization of CYP11A1 in the brain, we performed *in situ* hybridization (ISH) analyses on human cerebellum and cerebral cortex tissue slices using the RNAscope technology described by Wang *et al*. (38). A duplex chromogenic ISH assay using probes targeting *CYP11A1* or *MBP* was conducted. In the cerebellum, expression of *CYP11A1* was limited to a very small number of cells in the granule layer and molecular layer (Fig. 1, *D* and *E*). Interestingly, we found that a larger proportion of Purkinje cells had positive expression for *CYP11A1* (Fig. 1*E*). We could not observe the presence of *CYP11A1* mRNA in white matter of the cerebellum (Fig. 1*G*). Similar to the cerebellum, extremely low levels of *CYP11A1* mRNA were seen in the cerebral cortex gray matter with even lower expression in the white matter (Figs. 1*F* and S2). While the majority of the cortex white matter did not have detectable *CYP11A1* mRNA (Fig. S2, *E* and *G*), a very scarce number of cells showed positive *CYP11A1* signals in the cortex white matter of one donor (Fig. S2*H*). We estimate that the proportion of *CYP11A1*-positive cells is less than 1% of cells in both brain regions. Each *CYP11A1*-positive cell showed no more than two molecules of *CYP11A1* mRNA in our ISH assay. There was also little colocalization between *MBP* and *CYP11A1*, suggesting that oligodendrocytes and myelinated fibers are not likely to express the most *CYP11A1* in the cerebellum and cortex.

**Figure 2:**
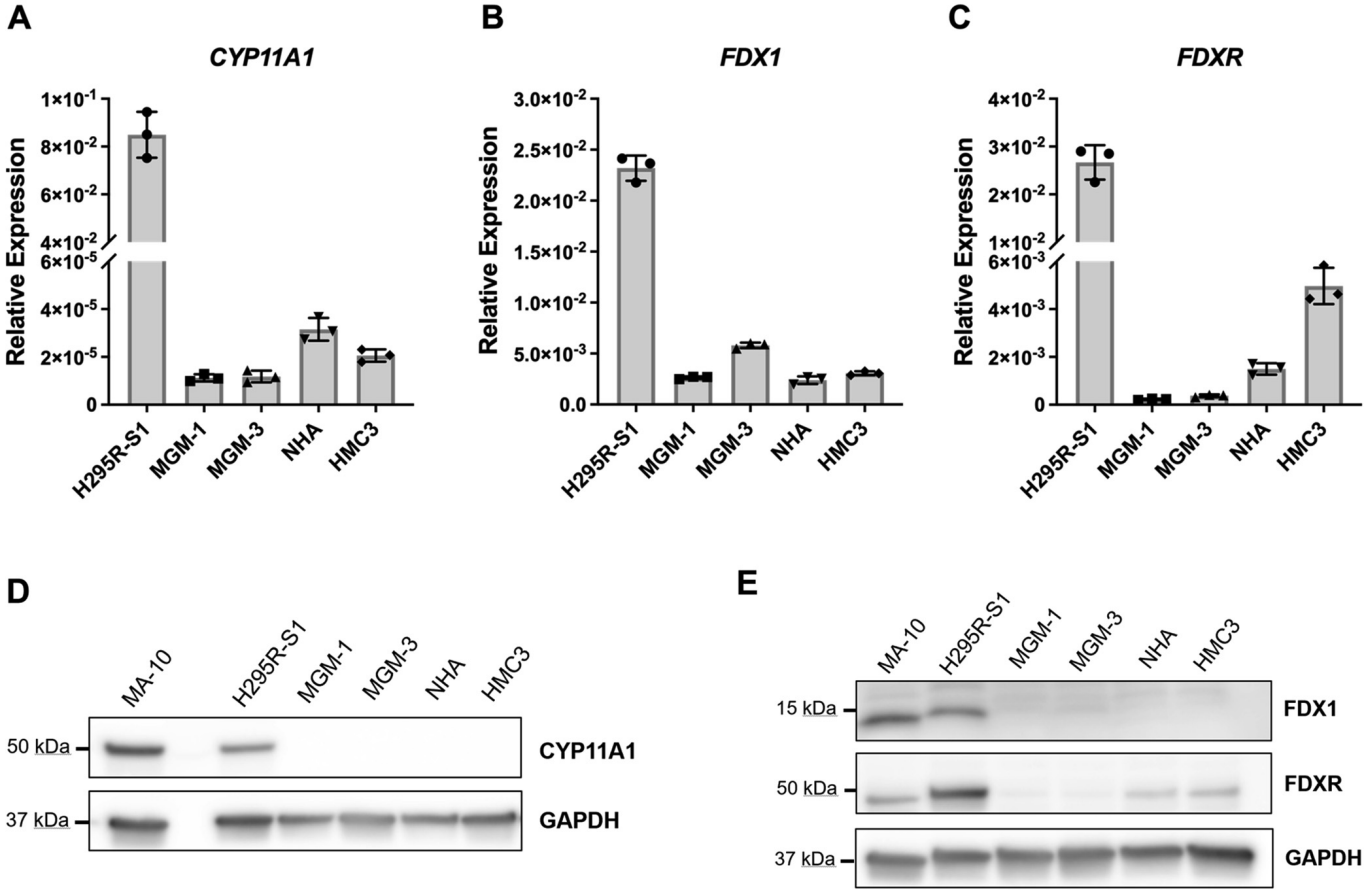
**RNA and protein expression of CYP11A1 and its cofactors in glial cells.***A*–*C* qRT-PCR analyses of CYP11A1, FDX1, and FDXR in human glial cells, with H295R-S1 as a positive control. Gene expression is shown as relative expression to α-tubulin. Data are presented as mean ± SD, N = 3. Each data point represents total RNA extracted from cells of a different passage for each cell line. *D* and *E* representative immunoblots of CYP11A1, FDX1, and FDXR in human glial cells, with MA-10 and H295R-S1 as positive controls. GAPDH was used as a loading control. The 15 μg of total cell lysate was loaded in each lane. No specific bands for CYP11A1 can be observed in glial cells. qRT-PCR, quantitative RT-PCR.

**Figure 3:**
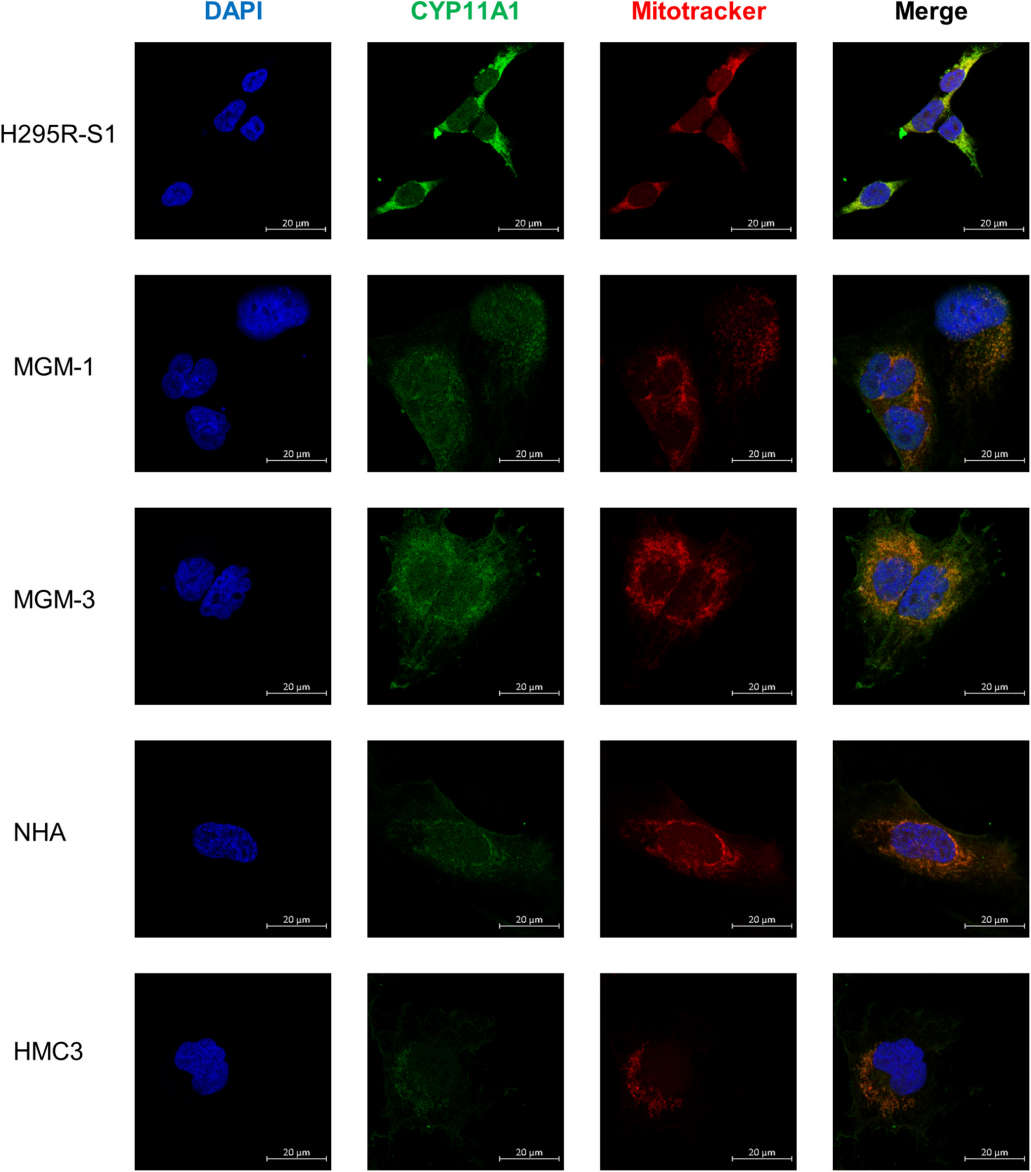
**Confocal images of H295R-S1, MGM-1, MGM-3, NHA, and HMC3 cells for CYP11A1 expression**. Cells stained with anti-CYP11A1 antibody (*green*), with mitochondria labeled with Mitotracker Red and nucleus stained with DAPI (*blue*). Images were taken at 63× magnification. H295R-S1 cells stain very strongly for CYP11A1, which overlaps entirely with mitochondrial staining. Very faint CYP11A1 staining colocalizing with mitochondria can be seen in MGM-1, MGM-3, NHA, and HMC3 cells, with the strongest signal in MGM-3 cells. DAPI, 4′,6-diamidino-2-phenylindole; NHA, normal human astrocyte.

Next, we measured *CYP11A1* expression in the glial cell lines (Fig. 2). All four glial cell lines expressed low but detectable amounts of *CYP11A1* mRNA, with relative expression between 10^3^ to 10^4^ times lower than H295R-S1 cells (Fig. 2*A*). We also confirmed that all four glial cell lines express mRNA for the CYP11A1 cofactors, *FDX1* and *FDXR* (Fig. 2, *B* and *C*). Despite the presence of low *CYP11A1* mRNA levels in the glial cells, we could not detect any CYP11A1 protein expression by immunoblotting using four different commercially available antibodies (Figs. 2*D* and S3, *A*–*D*). No specific signal for CYP11A1 could be observed on immunoblots even when up to 80 μg protein was loaded for glial cells (Fig. S3*E*). When protein bands around 40, 50, and 60 kDa were subjected to shotgun proteomics mass spectrometry (MS) analysis, no CYP11A1 protein/peptide sequences were found, confirming the lack of specific CYP11A1 bands in the immunoblots (dataset S1). Low levels of FDX1 and FDXR protein can be detected in the four glial cell lines (Fig. 2*E*). We were, however, able to detect low levels of CYP11A1 staining using immunofluorescence (Fig. 3). In glial cells, although the strongest punctate stains for CYP11A1 colocalized with mitochondria, the fluorescent signals were not clearly distinguishable from background fluorescence.

### Human glial cells synthesize pregnenolone that can be stimulated by TSPO ligand XBD-173 and hydroxycholesterols

**Figure 4:**
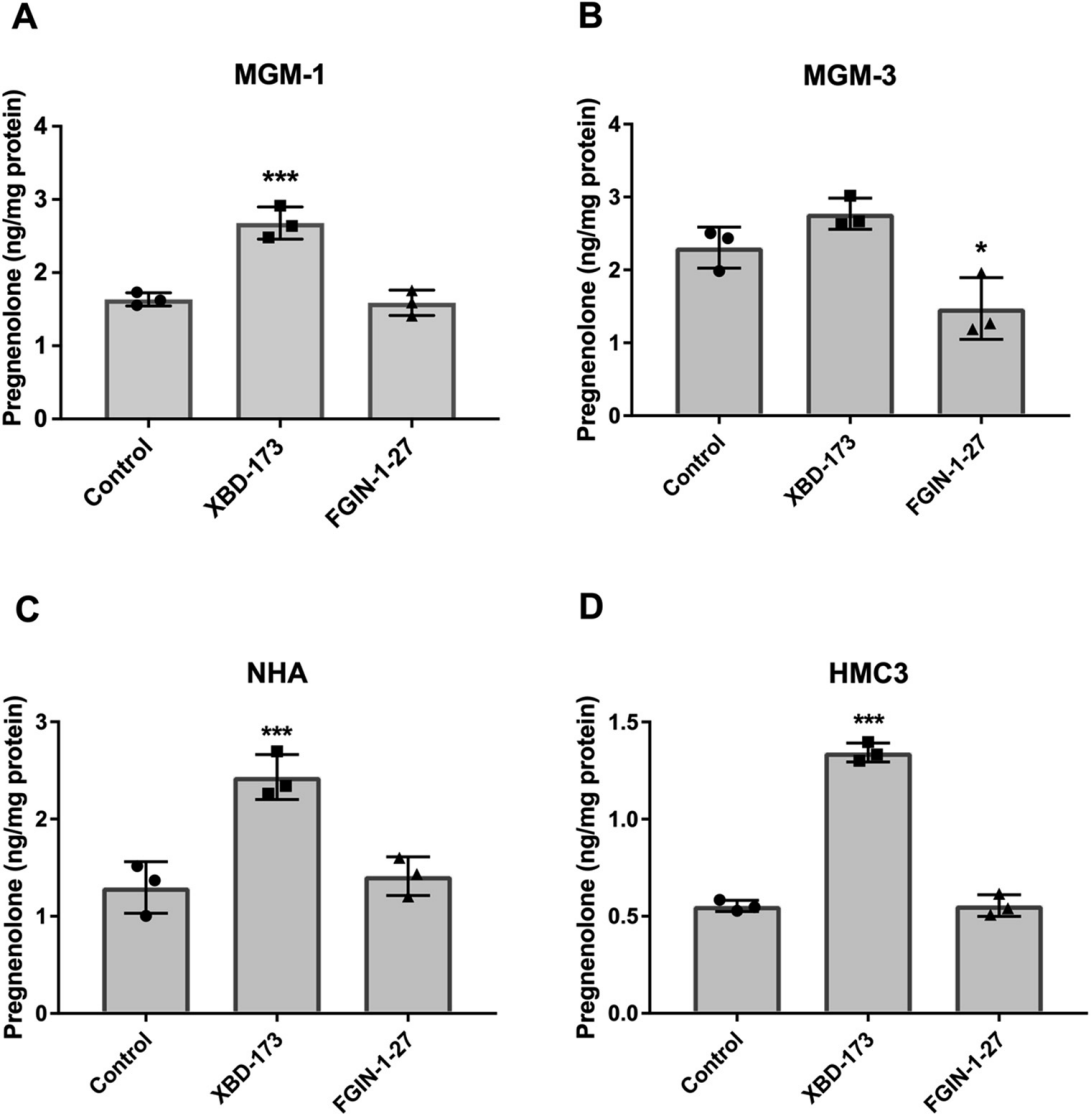
**Human glial cells produce pregnenolone and the production can be altered by TSPO ligands**. ELISA measurements of pregnenolone in culture media of MGM-1 (*A*), MGM-3 (*B*), NHA (*C*), and HMC3 (*D*) cells treated with DMSO control, 50 μM XBD-173, or 50 μM FGIN-1-27 for 2 h. Data are presented as mean ± SD, N = 3. Statistics performed compared to control group. Each data point represents the average of one experiment, where each treatment was performed in triplicate within each experiment. XBD-173 significantly stimulated pregnenolone production in MGM-1, NHA, and HMC3 cells (∗∗ *p* < 0.01, ∗∗∗ *p* < 0.001) but not MGM-3 cells. FGIN-1-27 inhibited pregnenolone production in MGM-3 cells (∗ *p* < 0.05) but had no effect in other glial cells. DMSO, dimethyl sulfoxide; NHA, normal human astrocyte.

**Figure 5:**
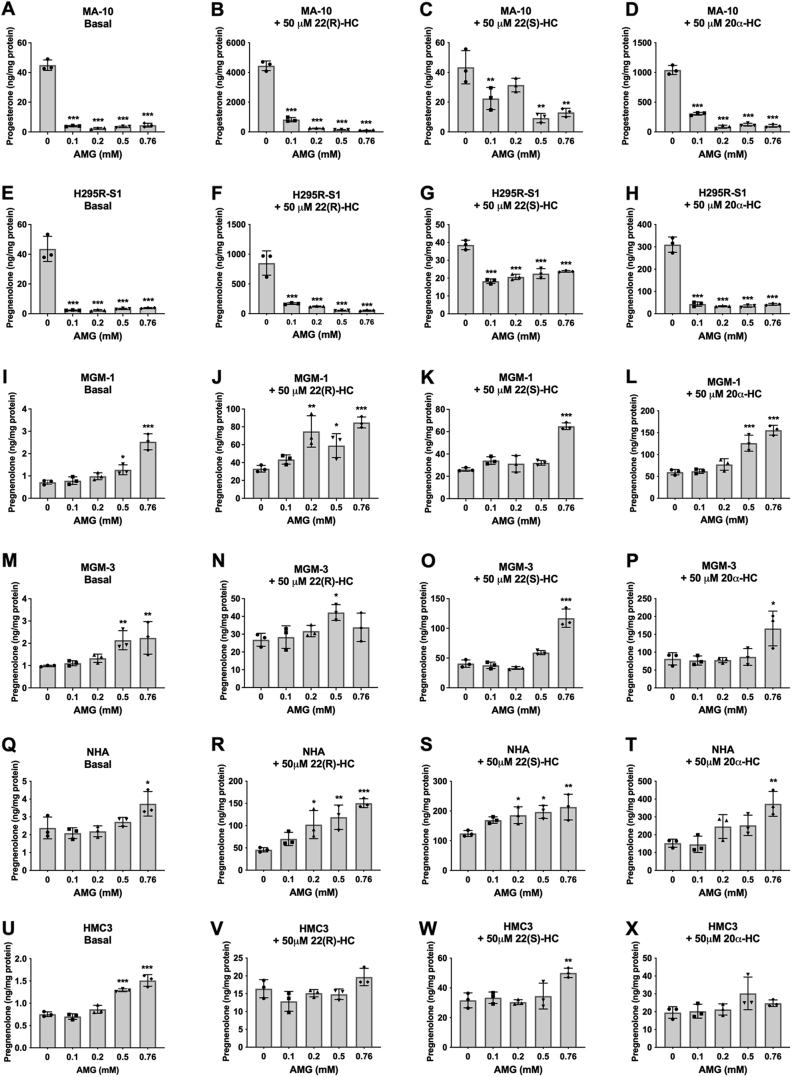
**Effect of aminoglutethimide (AMG) on basal and hydroxycholesterol-stimulated steroid production in peripheral cells and glial cells**. ELISA measurements of secreted progesterone by MA-10 cells (*A*–*D*) and secreted pregnenolone by H295R-S1 (*E*–*H*), MGM-1 (*I*–*L*), MGM-3 (*M*–*P*), NHA (*Q*–*T*), and HMC3 (*U*–*X*) cells when treated with four different doses of AMG for 2 h. Effects of AMG on secreted steroids under basal (*A*, *E*, *I*, *M*, *Q*, and *U*), 50 μM 22(R)-hydroxycholesterol stimulation (*B*, *F*, *J*, *N*, *R*, and *V*), 50 μM 22(S)-hydroxycholesterol stimulation (*C*, *G*, *K*, *O*, *S*, and *W*), and 50 μM 20α-hydroxycholesterol stimulation (*D*, *H*, *L*, *P*, *T*, and *X*) conditions are shown. Each data point represents the average of one experiment, where each treatment was performed in triplicate within each experiment. Data are presented as mean ± SD, N = 3. Statistics performed compared to 0 mM AMG group within each panel. AMG significantly inhibited steroid production in MA-10 and H295R-S1 cells under basal and hydroxycholesterol-stimulated conditions; however, AMG did not inhibit pregnenolone production in the four glial cell lines at low doses under basal and hydroxycholesterol-stimulated conditions. High doses of AMG increased pregnenolone production in glial cells. (∗ *p* < 0.5, ∗∗ *p* < 0.01, ∗∗∗ *p* < 0.001). NHA, normal human astrocyte.

Although CYP11A1 protein expression was barely detectable, we found that glial cells produced pregnenolone. Secreted pregnenolone in culture media was measured using ELISA and confirmed using MS (Fig. S4). In addition, pregnenolone levels accumulated in the culture media over time, confirming novel synthesis and secretion of pregnenolone by glial cells (Fig. S5, *A* and *B*). We found that pregnenolone production can be stimulated by the TSPO ligand XBD-173 but not the TSPO ligand FGIN-1-27 at 50 μM (Fig. 4). In MGM-3 cells, FGIN-1-27 inhibited pregnenolone production, but this trend was not observed in the other three glial cell lines. Pregnenolone production was also increased when cells were given CYP11A1 substrates, such as 22(R)-HC and 20α-hydroxycholesterol (20α-HC) (Fig. 5) (39). However, pregnenolone production was increased only in glial cells and not peripheral cells when cells were treated with 22(S)-hydroxycholesterol (22(S)-HC) (Fig. 5). This result suggests the presence of desmolase activity that can cleave the bond between C20 and C22 in hydroxycholesterols to produce pregnenolone. Taken together, these data indicate that human glial cells have the ability to synthesize pregnenolone and pregnenolone production can be stimulated by some of the compounds that increase steroid production in peripheral steroidogenic cells.

### Pregnenolone synthesis by glial cells is not inhibited by CYP11A1 inhibitors

To confirm whether an undetectable but small amount of CYP11A1 enzyme in glial cells is responsible for producing pregnenolone, we treated the cells with CYP11A1 inhibitors. The specific CYP11A1 inhibitor DL-aminoglutethimide (AMG) (40) significantly inhibited steroid production in a dose-dependent manner in H295R-S1 and MA-10 cells (Fig. 5, *A*–*H* and S6*A*). In the peripheral cells, AMG also significantly inhibited steroid formation in response to 22(R)-HC, 22(S)-HC, and 20α-HC treatment. However, in all four glial cell lines, AMG failed to inhibit steroid production at both the basal level and when induced with hydroxycholesterols (Fig. 5, *I*–*X* and S6*B*).

**Figure 6:**
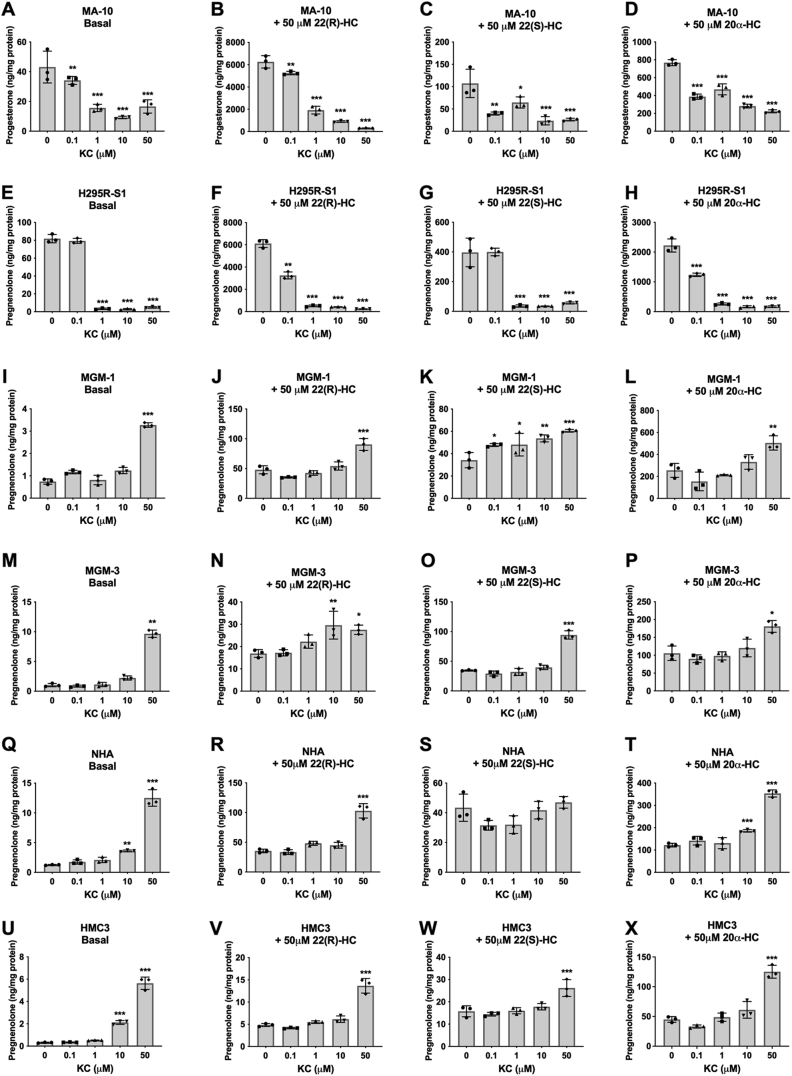
**Effect of ketoconazole (KC) on basal and hydroxycholesterol-stimulated steroid production in peripheral and glial cells**. ELISA measurements of secreted progesterone by MA-10 cells (*A*–*D*) and secreted pregnenolone by H295R-S1 (*E*–*H*), MGM-1 (*I*–*L*), MGM-3 (*M*–*P*), NHA (*Q*–*T*), and HMC3 (*U*–*X*) cells when treated with four different doses of KC for 2 h. Effects of KC on secreted steroids under basal (*A*, *E*, *I*, *M*, *Q*, and *U*), 50 μM 22(R)-hydroxycholesterol stimulation (*B*, *F*, *J*, *N*, *R*, and *V*), 50 μM 22(S)-hydroxycholesterol stimulation (*C*, *G*, *K*, *O*, *S*, and *W*), and 50 μM 20α-hydroxycholesterol stimulation (*D*, *H*, *L*, *P*, *T*, and *X*) conditions are shown. Each data point represents the average of one experiment, where each treatment was performed in triplicate within each experiment. Data are presented as mean ± SD, N = 3. Statistics performed compared to 0 μM KC group within each panel. KC significantly inhibited steroid production in H295R-S1 cells starting at 1 μM and in MA-10 cells starting at 0.1 μM. However, in glial cells, KC did not inhibit pregnenolone production at any concentration. In NHA and HMC3 cells, 10 μM KC increased pregnenolone secretion. In all glial cells, 50 μM KC increased pregnenolone secretion under almost all conditions. (∗ *p* < 0.5, ∗∗ *p* < 0.01, ∗∗∗ *p* < 0.001). NHA, normal human astrocyte.

**Figure 7:**
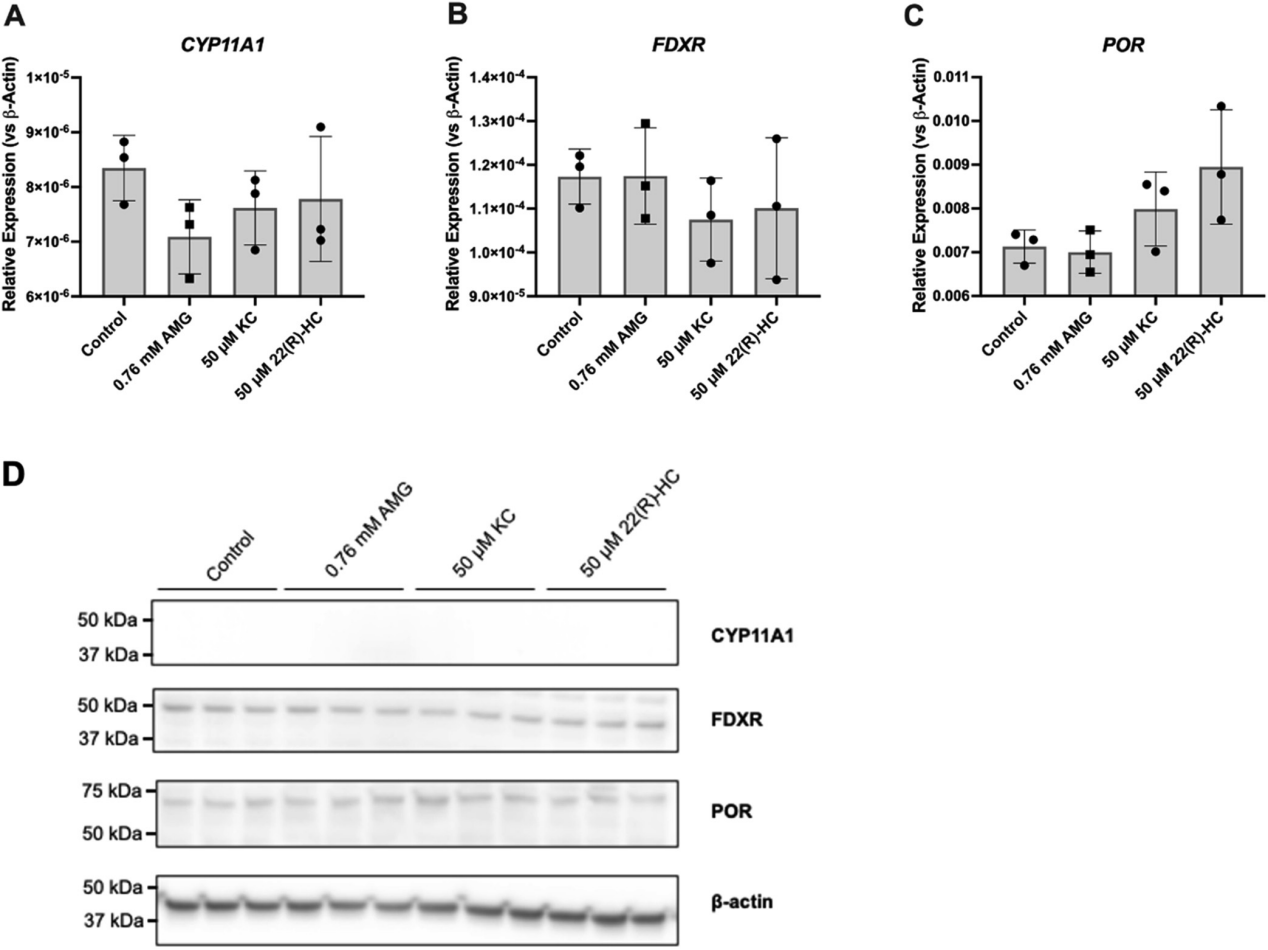
**Expression of CYP11A1, FDXR, and POR in MGM-1 cells after treatments that increase pregnenolone synthesis**. *A*–*C*, qRT-PCR analysis of *CYP11A1* expression (*A*) in addition to expression of CYP450 cofactors, *FDXR* (*B*) and *POR* (*C*), following treatment of 0.76 mM AMG, 50 μM ketoconazole (KC), or 50 μM 22(R)-hydroxycholesterol (22(R)-HC) for 2 h in MGM-1 cells. Gene expression is shown as relative expression to β-actin. Data are presented as mean ± SD, N = 3. *D*, representative immunoblot showing protein expression of CYP11A1, FDXR, and POR following the aforementioned treatments. β-Actin was used as a loading control. No significant changes were observed for either RNA or protein expression of CYP11A1, FDXR, and POR following AMG, KC, and 22(R)-HC treatments. AMG, aminoglutethimide; qRT-PCR, quantitative RT-PCR.

To confirm these results, we used a nonspecific inhibitor of CYP enzymes, ketoconazole (KC). KC inhibits multiple CYPs along the steroidogenic pathway—including CYP11A1, CYP17A1, CYP19A1, CYP11B1, and CYP11B2, as well as other CYPs such as CYP3A4 (41, 42). The effect of KC may indicate whether another CYP is responsible for pregnenolone synthesis. As expected, KC significantly inhibited steroid production in H295R-S1 and MA-10 cells (Fig. 6, *A*–*H*). However, similar to AMG, KC failed to inhibit steroid production in the four glial cell lines at basal and hydroxycholesterol-stimulated conditions (Fig. 6, *I*–*X*). A summary of these results is presented in Table 1. Interestingly, high doses of AMG and KC appeared to increase pregnenolone synthesis in glial cells. A 24 h time course study revealed that pregnenolone levels increased with time in cell supernatants after treatment of AMG or KC (Fig. S5, *C* and *D*), suggesting that these drugs stimulate pregnenolone production in glial cells rather than inhibiting it as in peripheral cells. This induced increase in pregnenolone secretion does not appear to be due to upregulation of CYP11A1 expression or upregulation of CYP450 cofactors (Fig. 7) but rather by another mechanism.

**Table 1:** Effects of AMG and KC on steroid synthesis

Cell line	Aminoglutethimide				Ketoconazole			
	Basal	+22(R)-HC	+22(S)-HC	+20α-HC	Basal	+22(R)-HC	+22(S)-HC	+20α-HC
**MA-10**	**↓**	**↓**	**↓**	**↓**	**↓**	**↓**	**↓**	**↓**
**H295R-S1**	**↓**	**↓**	**↓**	**↓**	**↓**	**↓**	**↓**	**↓**
**MGM-1**	**↑**	**↑**	**↑**	**↑**	**↑**	**↑**	**↑**	**↑**
**MGM-3**	**↑**	**↑**	**↑**	**↑**	**↑**	**↑**	**↑**	**↑**
**NHA**	**↑**	**↑**	**↑**	**↑**	**↑**	**↑**	**-**	**↑**
**HMC3**	**↑**	**-**	**↑**	**-**	**↑**	**↑**	**↑**	**↑**

↑ indicates a significant increasing and ↓ indicates a significant decreasing trend in steroid production. – indicates no significant change.

Our findings suggest that human brain cells can synthesize pregnenolone independently of CYP11A1 activity, and the apparent desmolase activity that allows synthesis of pregnenolone from hydroxycholesterols is not inhibited by CYP11A1 inhibitors AMG and KC. These findings support our recent discussion on the discrepancy between levels of CYP11A1 and pregnenolone found in the brain, where we suggested that alternative pathway(s) may be used to produce pregnenolone there (33). Using this hypothesis, we proposed three possibilities for the alternative pathway(s): (1) pregnenolone synthesis may be regulated by reactive oxygen species (ROS) in glial cells, (2) a second CYP11A1 isoform may be responsible for producing pregnenolone in the brain, and (3) another CYP450 enzyme other than CYP11A1 may be involved in synthesizing pregnenolone in the brain. These three possibilities will each be tested in the following sections.

### Pregnenolone synthesis in glial cells is not dependent on ROS

We first examined the involvement of ROS in pregnenolone synthesis by glial cells. Our results showed that high concentrations of the two CYP11A1 inhibitors AMG and KC seemed to increase rather than inhibit pregnenolone production in glial cells both at the basal and hydroxycholesterol-stimulated conditions. AMG and KC have also been reported to increase cellular ROS (43, 44). Furthermore, previous studies of steroidogenesis in MGM-1, MGM-3, and NHA cells have revealed that DHEA synthesis by these cells is stimulated by oxidative stress and is independent of CYP17A1 activity in the classical pathway (37). The dose-dependent stimulation in DHEA production by various oxidants was most apparent in MGM-1 cells. Thus, we postulated that pregnenolone may be synthesized in a ROS-dependent manner and tested this possibility in MGM-1 cells.

**Figure 8:**
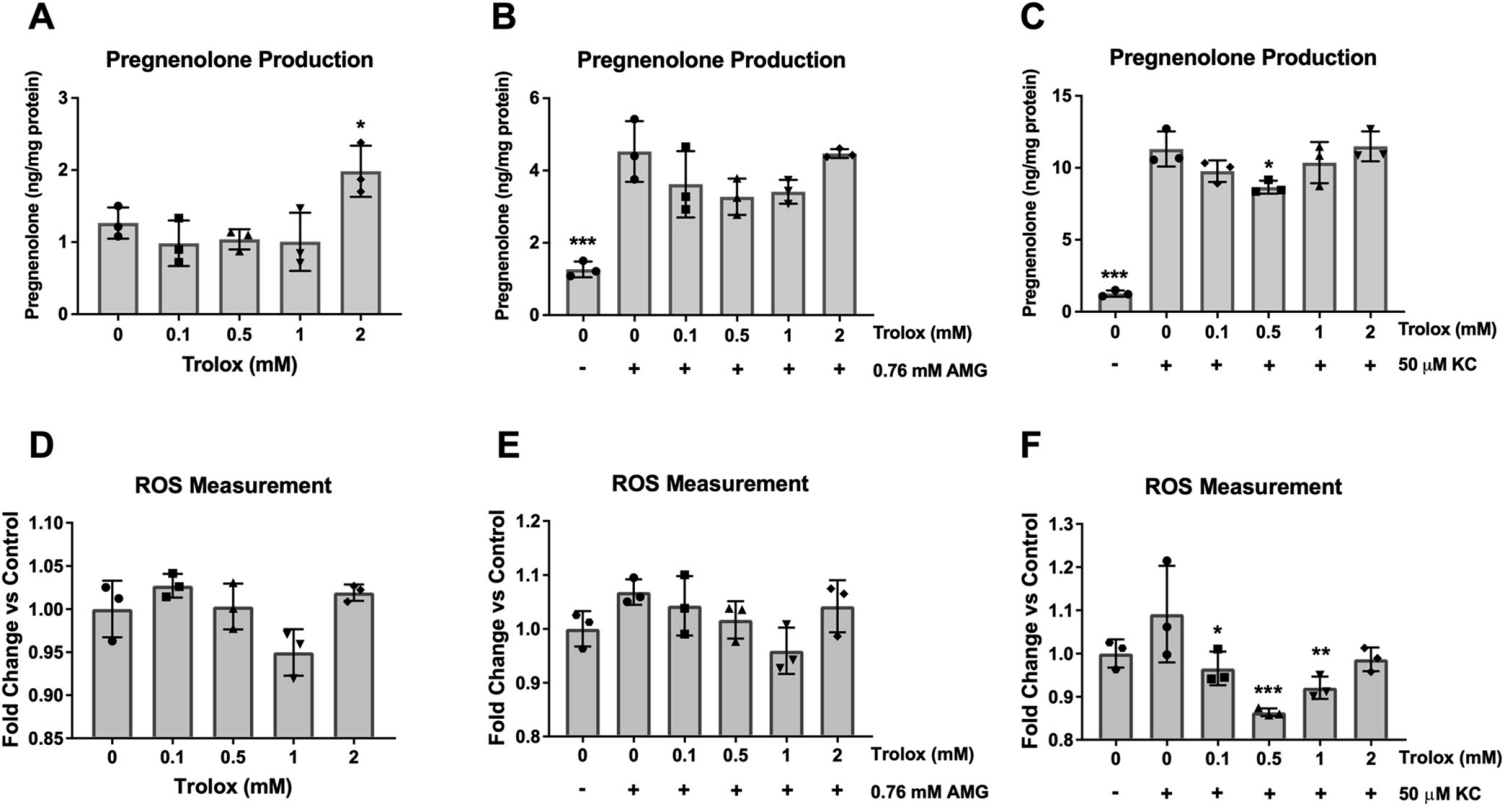
**Effect of antioxidant Trolox on pregnenolone secretion and intracellular ROS in MGM-1 cells**. ELISA measurements (*A*–*C*) of secreted pregnenolone and intracellular ROS measurements (*D*–*F*) when MGM-1 cells were treated with different doses of Trolox for 2 h, either alone (*A* and *D*) or combined with 0.76 mM AMG (*B* and *E*) or 50 μM KC (*C* and *F*). Each data point represents the average of one experiment, where each treatment was performed in triplicate within each experiment. Data are presented as mean ± SD, N = 3. Statistics performed compared to 0 mM Trolox (*A* and *D*), 0 mM Trolox + 0.76 mM AMG (*B* and *E*), or 0 mM Trolox + 50 μM KC (*C* and *F*). Trolox did not significantly change secreted pregnenolone or ROS except for a marginally significant increase in pregnenolone production with the 2 mM Trolox treatment. Although 0.76 mM AMG and 50 μM KC significantly increased pregnenolone production in MGM-1 cells, neither drug significantly altered intracellular ROS. Low doses of Trolox decreased ROS when MGM-1 cells were treated with 50 μM KC, but this effect was blunted at the highest dose of 2 mM Trolox. The trend seen with low dose Trolox combined with 50 μM KC did not correlate with changes in pregnenolone production. (∗ *p* < 0.05, ∗∗ *p* < 0.01, ∗∗∗ *p* < 0.001). AMG, aminoglutethimide; KC, ketoconazole; ROS, reactive oxygen species; Trolox, (±)-6-hydroxy-2,5,7,8-tetramethylchromane-2-carboxylic acid.

First, we examined the effects of the antioxidant (±)-6-hydroxy-2,5,7,8-tetramethylchromane-2-carboxylic acid (Trolox), an analog of vitamin E, on pregnenolone production and cellular ROS in MGM-1 cells (Fig. 8). We also cotreated the cells with high doses of AMG or KC to see whether an antioxidant could reduce their stimulatory effects on pregnenolone production. In MGM-1 cells, treatment with Trolox did not result in any significant changes in pregnenolone levels (Fig. 8*A*) or cellular ROS (Fig. 8*D*) overall. The increase in pregnenolone production with treatment of 2 mM Trolox was marginally significant (*p* = 0.0477). High dose AMG (0.76 mM) and high dose KC (50 μM) treatments led to higher pregnenolone production but no significant changes in cellular ROS (Fig. 8, *B*, *C*, *E* and *F*). Furthermore, the addition of Trolox had no significant effect on the increased pregnenolone production induced by AMG and KC (Fig. 8, *B* and *C*). Low doses of Trolox decreased the levels of cellular ROS induced by KC, but this effect was blunted at the highest dose of 2 mM (Fig. 8*F*). Trolox also did not induce significant changes in ROS when cells were cotreated with AMG (Fig. 8*E*). To confirm these results, we tested the same conditions in MGM-3 cells. Similar to MGM-1 cells, Trolox did not significantly affect pregnenolone synthesis and cellular ROS by itself or in combination with 0.76 mM AMG in MGM-3 cells (Fig. S8). On the other hand, 50 μM KC significantly increased cellular ROS in MGM-3 cells, and ROS induction was decreased by higher doses of Trolox (Fig. S8*F*). However, only lower doses of Trolox reduced the increase in pregnenolone when cells were treated with 50 μM KC (Fig. S8*C*). Therefore, our results indicate that antioxidants do not affect pregnenolone production in the glial cells.

**Figure 9:**
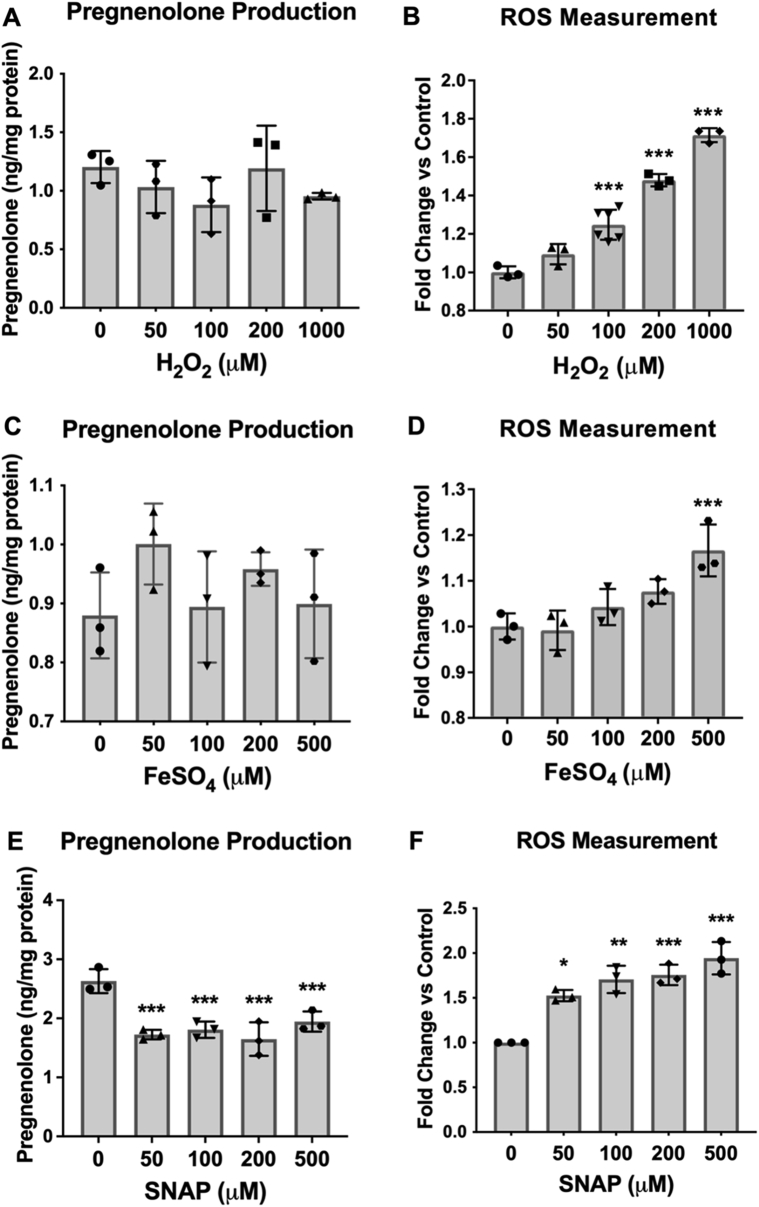
**Effect of oxidants on pregnenolone secretion and intracellular ROS in MGM-1 cells.** ELISA measurements (*A* and *C*) of secreted pregnenolone and intracellular ROS measurements (*B* and *D*) when MGM-1 cells were treated with different doses of hydrogen peroxide (H_2_O_2_; *A* and *B*) or ferrous sulfate (FeSO_4_; *C* and *D*). Each data point represents the average of one experiment, where each treatment was performed in triplicate within each experiment. Data are presented as mean ± SD, N = 3. Statistics performed compared to the no treatment group within each panel. Hydrogen peroxide treatment significantly increased intracellular ROS starting at 100 μM but did not alter pregnenolone secretion by MGM-1 cells. Ferrous sulfate treatment did not significantly increase intracellular ROS except at 500 μM and did not change pregnenolone production by MGM-1 cells. (∗ *p* < 0.05, ∗∗ *p* < 0.01, ∗∗∗ *p* < 0.001). ROS, reactive oxygen species.

Since antioxidant treatment failed to induce significant changes in pregnenolone synthesis, we aimed to confirm the role of ROS in pregnenolone synthesis by treating MGM-1 cells with two oxidants: hydrogen peroxide (H_2_O_2_) and ferrous sulfate (FeSO_4_). These two oxidants were chosen because they were previously shown to increase pregnenolone production in C6 rat glial cells, with 10 mM H_2_O_2_ inducing a twofold increase and 10 mM FeSO_4_ inducing a 3.6-fold increase (45). However, in MGM-1 cells, H_2_O_2_ had no significant effect on pregnenolone production (Fig. 9*A*), despite significantly increasing cellular ROS starting at 100 μM (Fig. 9*B*). We also found that FeSO_4_ did not have any significant effects on pregnenolone synthesis (Fig. 9*C*). However, after 2 h of FeSO_4_ treatment at 500 μM, the highest dose, there was a significant increase in cellular ROS, with a nonsignificant increasing trend at lower concentrations (Fig. 9*D*). In our preliminary studies where we pretreated MGM-1 cells with FeSO_4_ for 24 h, we had observed a significant increase in ROS starting at 50 μM but there were still no significant changes in pregnenolone production (data not shown). The same results were seen in MGM-3 cells where both H_2_O_2_ and FeSO_4_ failed to induce significant changes in pregnenolone production (Fig. S9).

To confirm whether other forms of oxidative stress may be involved in pregnenolone synthesis, we treated MGM-1 cells with the nitric oxide donor S-nitroso-N-acetylpenicillamine (SNAP). Interestingly, SNAP significantly inhibited pregnenolone production by the cells (Fig. 9*E*), despite dose-dependently increasing cellular ROS levels the same as other oxidants we tested (Fig. 9*F*). Given that there is no correlation between changes in intracellular ROS levels and pregnenolone production, we believe that the inhibitory effects of nitric oxide on pregnenolone synthesis are due to another mechanism, which will be discussed further in a later section. Taken together, our antioxidant and oxidant experimental results provide evidence against our first possible pathway; thus, the alternative pathway for pregnenolone synthesis does not appear to be dependent on ROS.

### Overexpression of CYP11A1b did not alter pregnenolone secretion unlike overexpression of CYP11A1a

Next, we examined the roles of different CYP11A1 isoforms in pregnenolone synthesis. A second CYP11A1 isoform has been reported in mouse and humans (33), but the function, expression, and localization of this isoform has not yet been studied. For isoform a (*i.e.*, the classical CYP11A1 enzyme), CYP11A1a will be used to denote the protein while *CYP11A1* variant 1 will be used to indicate the mRNA transcript Accessed Apr 11, 2022. https://www.ncbi.nlm.nih.gov/gene/1583. Similarly, CYP11A1b will be used to denote the protein for isoform b and *CYP11A1* variant 2 will be used to indicate the mRNA transcript. In comparison to isoform a, isoform b is 158 aa shorter from the N terminus. This second isoform could potentially be resistant to the inhibitory effects of AMG and KC due to possible structural differences but may still be able to produce pregnenolone. Therefore, human glial cells may use the second CYP11A1 isoform to synthesize pregnenolone.

**Figure 10:**
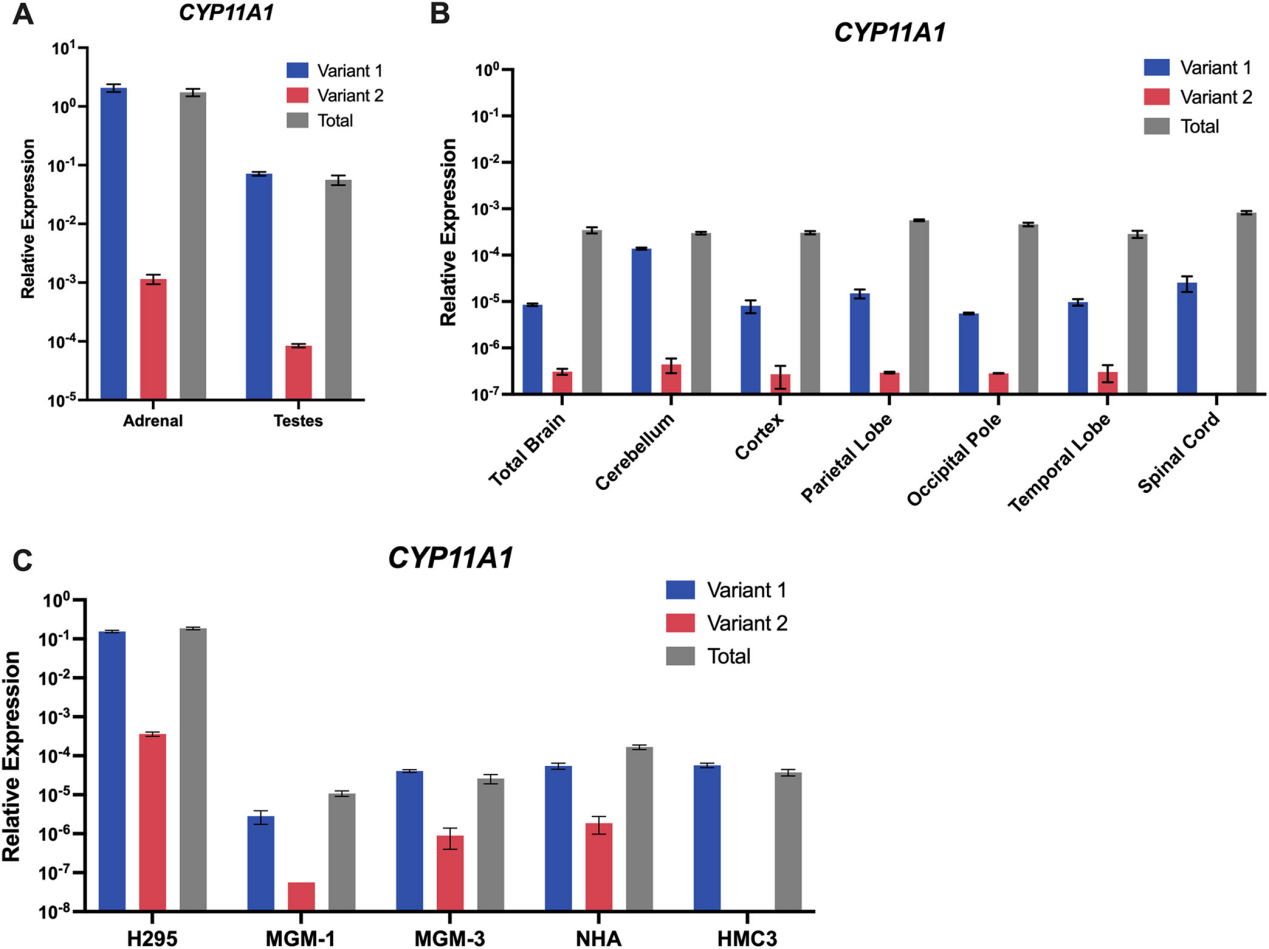
**RNA Expression of *CYP11A1* variants in the human CNS and peripheral tissues and cells**. qRT-PCR analyses of total *CYP11A1* (*gray*), *CYP11A1* variant 1 (*blue*), and *CYP11A1* variant 2 (*red*) expression in human peripheral steroidogenic tissues (*A*), human CNS tissues (*B*), and cell lines (*C*). Gene expression is shown as relative expression to β-actin. Data are presented as mean ± SD. CNS, central nervous system; qRT-PCR, quantitative RT-PCR.

We first examined the levels of the *CYP11A1* variants in the human brain and in brain cells (Fig. 10). Since the mRNA transcripts differ only by the sequence of exon 1, we conducted qRT-PCR to compare the expression of the variants using primers designed for exon 1 from each respective variant. The total *CYP11A1* expression was determined using primers complementary to sequences from the common exons. In peripheral steroidogenic organs and H295R-S1 adrenal cells, it was very clear that *CYP11A1* variant 1 is the predominant variant and makes up almost the entirety of the total *CYP11A1* expression (Fig. 10, *A* and *C*). Although the relative expression of *CYP11A1* variant 2 appears to be lower than that of *CYP11A1* variant 1 in all CNS tissues and brain cells, the total *CYP11A1* expression was higher than the summed expression of the variants except for in MGM-3 and HMC3 cells (Fig. 10, *B* and *C*). This may suggest an early truncation of the RNA transcript for CYP11A1b, since exon 1 is not translated into the final protein for this isoform. We could not accurately determine the protein levels for CYP11A1b in the human brain cells because CYP11A1b does not have a unique amino acid sequence that can differentiate it from CYP11A1a, and commercial antibodies that only bind CYP11A1a did not produce clear bands on immunoblots for glial cells to allow for subtraction (Fig. S3, *A* and *B*).

**Figure 11:**
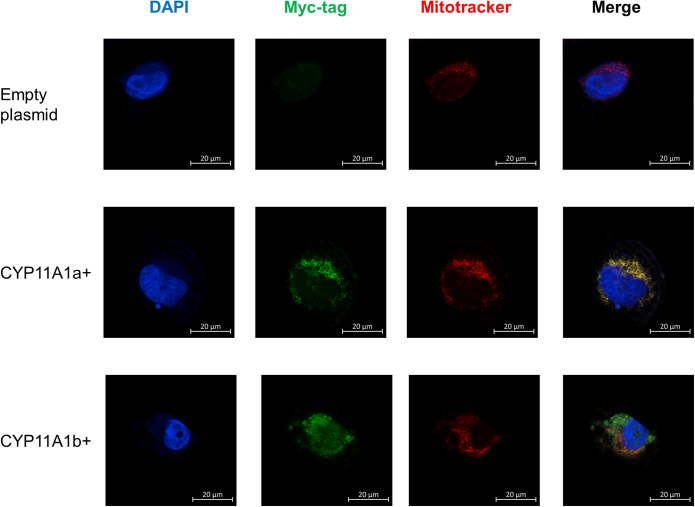
**Localization of CYP11A1 isoforms.** Representative confocal images of MGM-1 cells transfected with CYP11A1a-myc-DDK or CYP11A1b-myc-DDK expression vector. Cells transfected with empty plasmid were used as a negative control. Cells were stained with anti-myc-tag antibody (*green*), with mitochondria labeled with Mitotracker Red and nuclei stained with DAPI (*blue*). Images were taken at 63× magnification. CYP11A1a is strictly localized to the mitochondria. CYP11A1b appears more dispersed throughout the cell and has limited colocalization with mitochondria. DAPI, 4′,6-diamidino-2-phenylindole.

**Figure 12:**
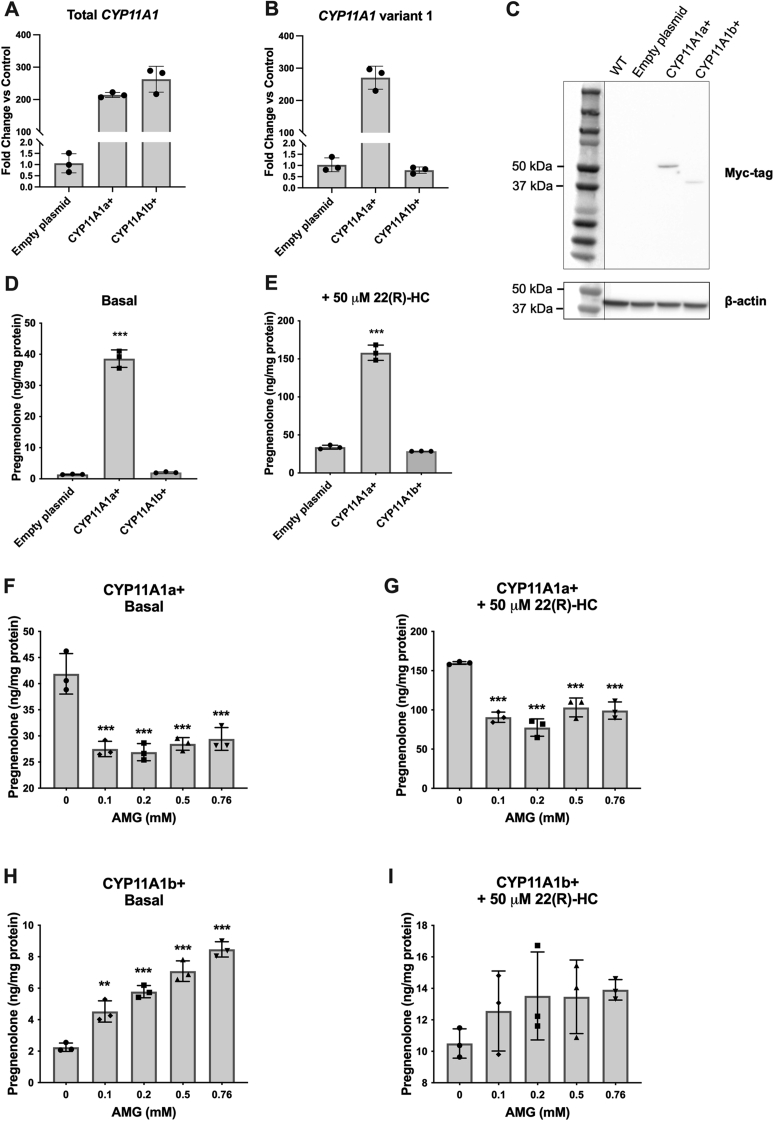
**CYP11A1a but not CYP11A1b overexpression leads to increased pregnenolone production that can be inhibited by AMG**. *A* and *B*, qRT-PCR analysis of *CYP11A1* expression in MGM-1 cells transfected with empty plasmid (negative control) or expression vectors for CYP11A1a or CYP11A1b with Myc-DDK tag. Gene expression is shown as fold change *versus* negative control, normalized to the expression of β-actin. Data are presented as mean ± SD, N = 3. Each data point represents total RNA extracted from pooled cells from different passages. *A*, transfected MGM-1 cells showed more than 200-fold increase in total *CYP11A1* mRNA expression. *B*, expression changes of *CYP11A1* variant 1 exon 1 indicate that the correct CYP11A1 isoform had been expressed. *C*, representative immunoblots for myc-tag in transfected MGM-1 cells. β-Actin was used as a loading control. Thirty micrograms of protein were loaded into each lane. A band corresponding to CYP11A1a was observed at around 50 kDa and a band corresponding to CYP11A1b was observed at around 42 kDa. No specific bands could be observed in WT or transfection control MGM-1 cells. *D* and *E*, ELISA measurements of pregnenolone secreted by transfected MGM-1 cells, with or without 22(R)-hydroxycholesterol stimulation. *F*–*I*, ELISA measurements of pregnenolone secreted by transfected MGM-1 cells treated with different doses of AMG for 2 h under basal (*F* and *H*) and 22(R)-hydroxycholesterol stimulated (*G* and *I*) conditions. Each data point represents the average of one experiment, where each treatment was performed in triplicate within each experiment. Data are presented as mean ± SD, N = 3. Statistics performed compared to control (*D* and *E*) or to 0 mM AMG group (*F*–*I*). CYP11A1a+ cells synthesized significantly more pregnenolone than controls and CYP11A1b+ cells, which were significantly inhibited by AMG. CYP11A1b+ cells behaved similarly to WT MGM-1 cells for pregnenolone synthesis. (∗∗ *p* < 0.01, ∗∗∗ *p* < 0.001). AMG, aminoglutethimide; qRT-PCR, quantitative RT-PCR.

To characterize the CYP11A1 isoforms better, we transfected MGM-1 cells with a CYP11A1a or CYP11A1b expression vector with a myc-DDK tag. After drug selection and colony expansion, we verified that the transfected MGM-1 cells highly expressed the corresponding CYP11A1 isoforms (Figs. 11 and 12, *A*–*C*). As expected, CYP11A1a is strictly localized to the mitochondria while CYP11A1b has a more diffused localization throughout the cell and limited colocalization with mitochondria (Fig. 11). The *CYP11A1* variant 2 expression vector used did not contain the unique exon 1 sequence as it is not translated into the final protein; thus, successful transfection was observed through increased total *CYP11A1* expression without an increase in *CYP11A1* variant 1 exon 1 (Fig. 12, *A* and *B*). CYP11A1b protein has a molecular weight of approximately 42 kDa on immunoblots while CYP11A1a appears as an approximately 50 kDa protein (Fig. 12*C*).

Cells overexpressing CYP11A1a showed approximately a 27-fold increase in basal pregnenolone production and a threefold increase in 22(R)-HC-stimulated pregnenolone production compared to control cells (Fig. 12, *D* and *E*). Unlike WT MGM-1 cells where AMG had no inhibitory effect (Fig. 5, *I* and *J*), pregnenolone production in CYP11A1a+ cells was significantly inhibited by AMG under both basal and hydroxycholesterol-stimulated conditions (Fig. 12, *F* and *G*). These results suggest that overexpressing the classical CYP11A1a in MGM-1 cells significantly increases pregnenolone synthesis. This enhanced production can be inhibited by AMG similar to adrenal H295R-S1 and MA-10 Leydig cells that use the classical steroidogenesis pathway. These data imply that MGM-1 cells are not inherently resistant to the effects of AMG, but rather endogenous pregnenolone production by MGM-1 cells is independent of classical CYP11A1a. On the other hand, overexpression of CYP11A1b did not significantly alter pregnenolone production compared to control cells (Fig. 12, *D* and *E*), and the effects of AMG on CYP11A1b+ cells were similar to that of WT cells, where AMG failed to inhibit pregnenolone synthesis and instead increased pregnenolone levels at higher doses (Fig. 12, *H* and *I*). These results indicate that CYP11A1b is not involved in pregnenolone production unlike CYP11A1a and that the second possibility listed previously of another isoform involved in pregnenolone production is unlikely.

### The alternative pathway for pregnenolone synthesis likely involves another CYP

**Figure 13:**
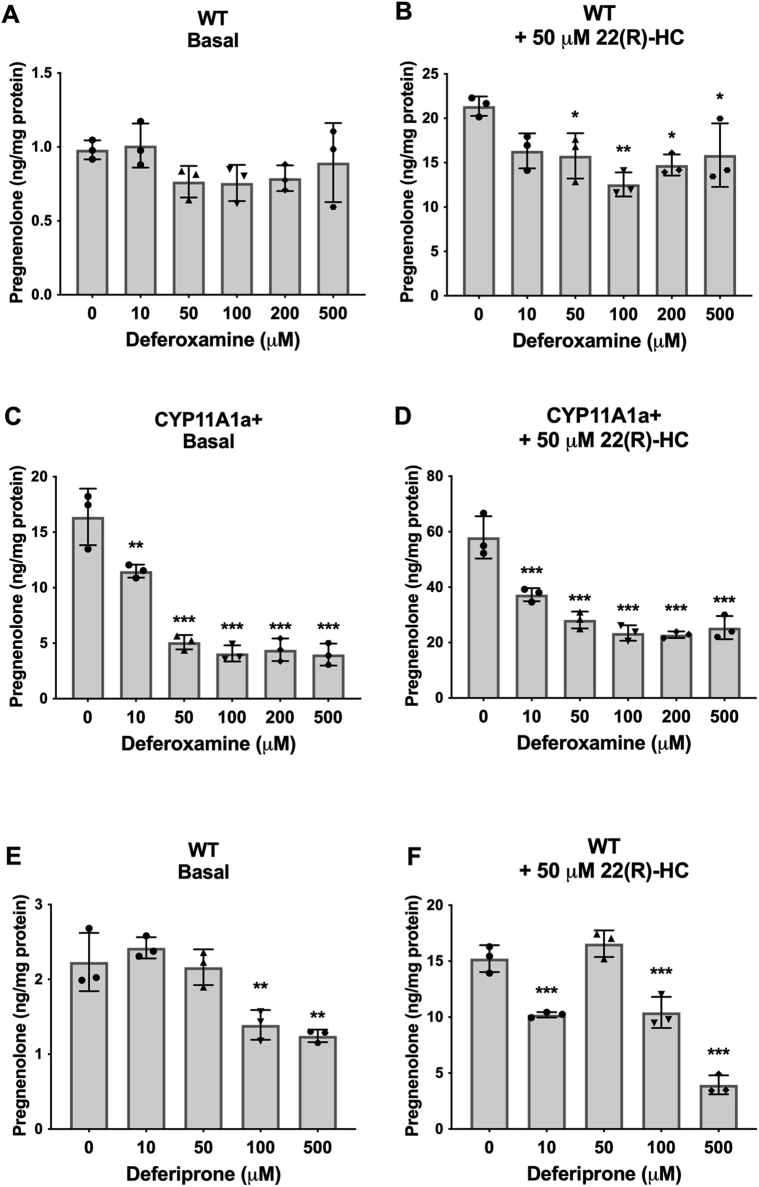
**Effect of iron chelation on pregnenolone secretion in MGM-1 cells**. ELISA measurements of secreted pregnenolone when MGM-1 WT (*A*, *B*, *E*, and *F*) and CYP11A1a+ (*C* and *D*) cells were pretreated with different doses of deferoxamine (*A*–*D*) or deferiprone (*E*–*F*) for 24 h. Each data point represents the average of one experiment, where each treatment was performed in triplicate within each experiment. Data are presented as mean ± SD, N = 3. Statistics performed compared to the no treatment group within each panel. Deferoxamine significantly inhibited pregnenolone production in CYP11A1+ cells and 22(R)-HC stimulated WT cells but had no statistically significant effect on WT cells under basal conditions. Deferiprone significantly inhibited pregnenolone production at high doses in basal and 22(R)-HC stimulated WT cells. (∗ *p* < 0.05, ∗∗ *p* < 0.01, ∗∗∗ *p* < 0.001). 22(R)-HC, 22(R)-hydroxycholesterol.

We demonstrated previously that the nitric oxide donor SNAP significantly inhibited pregnenolone synthesis. Given that there was no correlation between changes in intracellular ROS and pregnenolone production, we propose that the effect of SNAP on pregnenolone synthesis may be due to inhibition of CYP activity (46). Since CYPs rely on iron redox reactions for their activities, we tested the effects of iron chelation on pregnenolone synthesis. In WT MGM-1 cells, deferoxamine inhibited pregnenolone production, but this effect was only statistically significant when cells were stimulated with 22(R)-HC (Fig. 13, *A* and *B*). In CYP11A1a+ MGM-1 cells, deferoxamine inhibited pregnenolone production in a dose-dependent manner under both basal and 22(R)-HC stimulated conditions, demonstrating effective inhibition of CYP450 activity (Fig. 13, *C* and *D*). To confirm the effects of iron chelation on pregnenolone synthesis, we treated the cells with another but structurally different iron chelator, deferiprone (47). Indeed, pregnenolone production was significantly inhibited by deferiprone at high doses in MGM-1 cells (Fig. 13, *E* and *F*). The inhibitory effects of SNAP and iron chelation on pregnenolone secretion by MGM-1 cells suggest that the alternative pathway for pregnenolone synthesis may involve activity of another CYP450.

**Figure 14:**
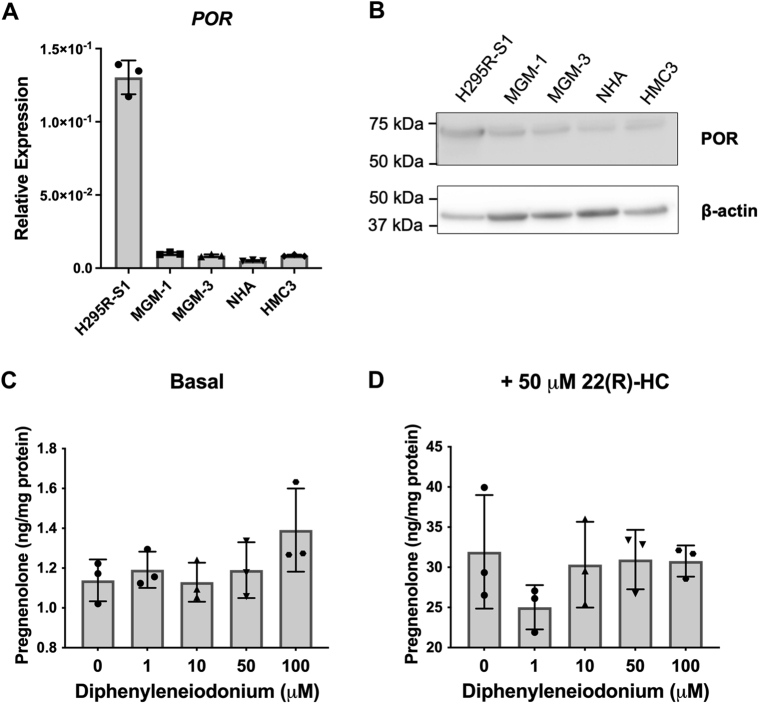
**POR expression in brain cells and effect of POR inhibition on pregnenolone synthesis**. *A*, qRT-PCR analysis of *POR* expression in human cell lines. Gene expression is shown relative to β-actin. *B*, representative immunoblot of POR in human cell lines, with β-actin as a loading control. Forty micrograms total protein were loaded into each lane. *C* and *D*, ELISA measurements of pregnenolone levels in culture media after MGM-1 cells were pretreated with different doses of diphenyleneiodonium for 24 h under both basal (*C*) and 22(R)-HC stimulated (*D*) conditions. Data are presented as mean ± SD, N = 3. Statistics performed compared to the no treatment group. No significant changes in pregnenolone production were found after diphenyleneiodonium treatment. 22(R)-HC, 22(R)-hydroxycholesterol; qRT-PCR, quantitative RT-PCR.

To further test this possibility, we explored the effects of inhibiting CYP450 cofactors, specifically POR and FDXR. CYP450 enzymes in the endoplasmic reticulum accept electrons from NADPH *via* POR while enzymes in the mitochondria accept electrons *via* FDXR and FDX1 (27). Therefore, the effects of inhibiting POR and FDXR may confirm whether the alternative pathway for pregnenolone synthesis is indeed dependent on CYP450 activity and determine the localization of the particular CYP450 involved. We first ascertained the expression of POR in the cell lines. Although H295R-S1 adrenal cells have higher POR expression than glial cells (Fig. 14, *A* and *B*), RNA expression of *POR* in glial cells is higher than that of *FDXR* (Figs. 14*A* and 2*C*), and POR protein is clearly detectable by immunoblot (Figs. 14*B* and S10*A*). However, treatment with diphenyleneiodonium, an inhibitor of POR, failed to alter pregnenolone production in glial cells under both basal and 22(R)-HC stimulated conditions (Fig. 14, *C* and *D*).

**Figure 15:**
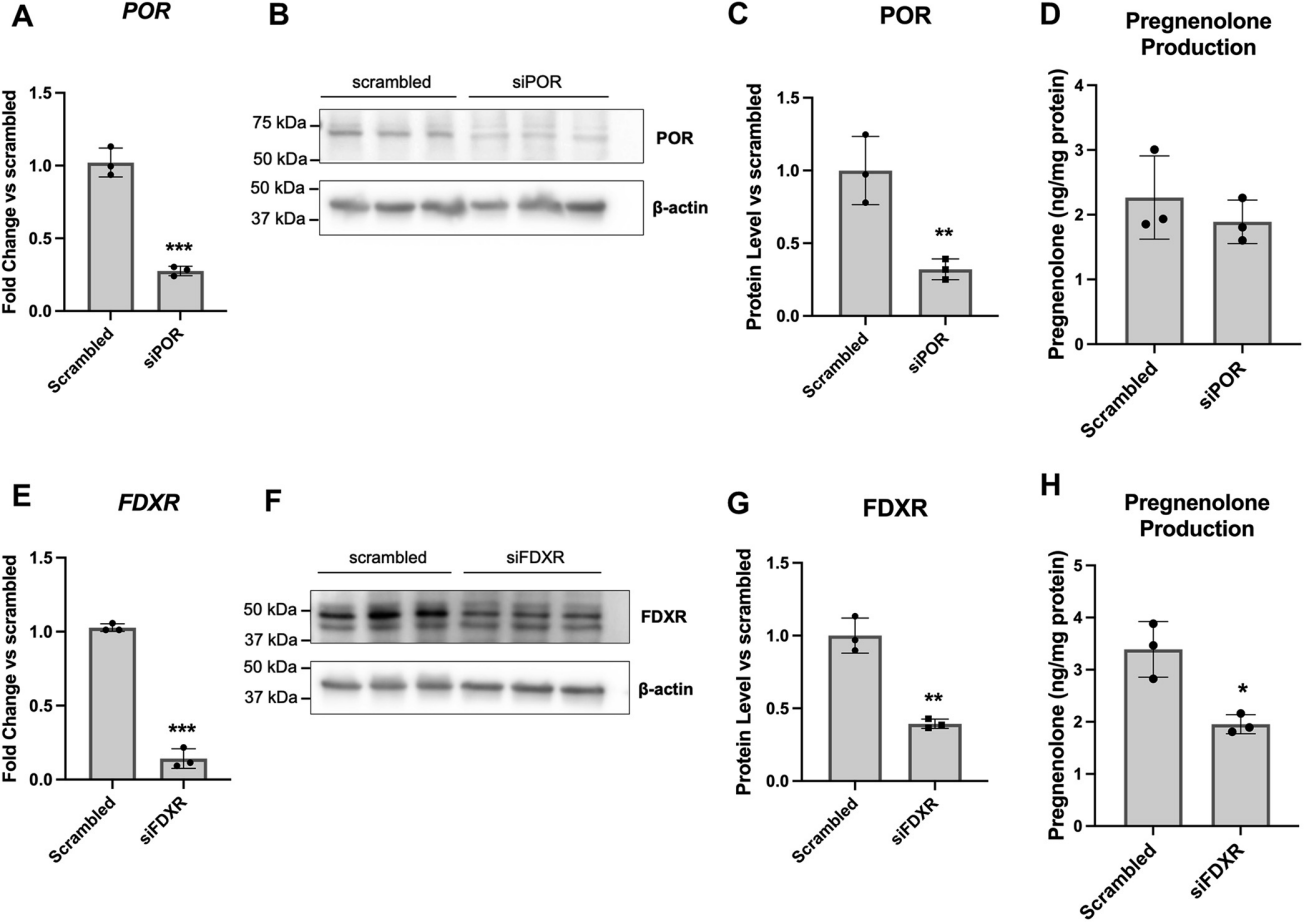
**Effects of POR and FDXR knockdown on pregnenolone synthesis in MGM-1 cells**. siRNA knockdown of POR (*A*–*D*) or FDXR (*E*–*H*) in MGM-1 cells. Knockdown efficiency was determined by qRT-PCR (*A* and *E*) and immunoblot (*B*, *C*, *F*, and *G*), with expression normalized to β-actin. *B* and *F*, representative immunoblots indicating successful knockdown. Twenty micrograms total protein were loaded into each lane. *C* and *G* quantification of protein levels in immunoblots in all experiments. *D* and *H* ELISA measurements of secreted pregnenolone in transfected MGM-1 cells. Data are presented as mean ± SD, N = 3. Statistics performed using unpaired *t* test. (∗ *p* < 0.05, ∗∗ *p* < 0.01, ∗∗∗ *p* < 0.001). qRT-PCR, quantitative RT-PCR.

These data appear to suggest that pregnenolone synthesis in glial cells is not dependent on POR and thus not dependent on a CYP450 activity in the endoplasmic reticulum. However, in addition to POR, diphenyleneiodonium also inhibits other enzymes such as NADPH oxidase, nitric oxide synthase, and xanthine oxidase, which have more varied subcellular localizations including the cytoplasm, cytoskeleton, vesicular organelles, nuclei, and other organelles depending on cell type (48, 49, 50, 51). Therefore more specific inhibition of POR would be needed to confirm its lack of involvement in pregnenolone synthesis. Thus, we performed siRNA knockdown of POR in MGM-1 cells and compared its effect to FDXR knockdown (Fig. 15). We were able to achieve >60% knockdown of both POR and FDXR (Fig. 15, *A*–*C* and *E*–*G*). Knockdown of POR failed to alter pregnenolone synthesis (Fig. 15*D*), supporting the results observed with diphenyleneiodonium. On the other hand, knockdown of FDXR significantly decreased pregnenolone synthesis by MGM-1 cells (Fig. 15*H*). Altogether, our results suggest that our third possibility is most probable: the alternative pathway for pregnenolone production in the brain involves a CYP450 enzyme activity other than CYP11A1 and the responsible enzyme(s) is likely localized to the mitochondria as they are dependent on FDXR activity.

## Discussion

We found *CYP11A1* mRNA in various parts of the human brain and four human glial cell lines, with levels between 1000 to 10,000 times lower than that in human adrenal cells and tissue. The difference in expression levels agrees with previous studies comparing *CYP11A1* mRNA levels between human brain and adrenal tissues (32, 52). The expression for *FDX1* and *FDXR* is significantly higher than *CYP11A1* in the glial cells, suggesting that any present CYP11A1 activity would not be limited by availability of cofactors. Although the microglia cell line HMC3 appears to have the highest expression of FDXR compared to the other glial cell lines, it produces the least amount of pregnenolone. HMC3 cells also have the lowest mRNA expression for most steroidogenic enzymes compared to the other glial cell lines, which indicates that microglia are not likely the main steroidogenic cell type in the human brain. Interestingly, although it is believed that steroidogenesis is involved in the modulation of inflammatory responses by microglia due to upregulation of TSPO during neuroinflammation (53), very few studies have measured steroid production in human microglia. In fact, some studies reported the lack of CYP11A1 in mouse and human microglia (54, 55). TSPO ligand XBD-173 was shown to increase pregnenolone synthesis in mouse microglia cell line BV-2 (56), but it failed to induce pregnenolone production in the human microglia cell line C20 (57). In contrast, our results here show that the human microglia cell line HMC3 can produce pregnenolone and production can be enhanced by both XBD-173 and hydroxycholesterols. The discrepancy between our results and those of Milenkovic *et al*. may be due to the higher concentration of XBD-173 and shorter stimulation period to prevent desensitization used in our study, as well as differences in the cell lines. Nevertheless, our results provide additional evidence that human microglia are indeed steroidogenic.

Despite many studies, including our current one, showing the presence of mRNA for *CYP11A1* in the human brain, there is a lack of studies demonstrating CYP11A1 protein in this tissue. The only two reports showing CYP11A1 protein in the human brain found very limited positive immunohistochemical staining in the prefrontal cortex (35) and cerebellum (34). Our ISH data revealed that *CYP11A1* mRNA is present in less than 1% of cells in the cortex and cerebellum, with each positive cell having no more than two mRNA molecules. *CYP11A1*-positive cells were mostly in the gray matter of the cerebellum and cerebral cortex, as well as the Purkinje cell layer in the cerebellum. In contrast to previous rat studies that found CYP11A1 protein localization in the white matter of various brain regions (58, 59), we observed very little *CYP11A1*-positive cells in the white matter. With such a low mRNA concentration for *CYP11A1* in the human brain, it is unlikely that CYP11A1 protein would be detectable in human brain tissue without using ultrasensitive methods.

Previous studies have found weak immunocytochemical staining of CYP11A1 in MGM-1, MGM-3, and NHA cells (37). We obtained similar results using immunofluorescence, with MGM-3 showing the highest CYP11A1 protein expression among the glial cell lines. However, we could not detect any specific bands for CYP11A1 using up to 80 μg of total cell lysate in immunoblotting for all four glial cell lines. Considering the detection limit of our immunoblotting system, which allows identification of signals from >0.3 ng protein, we estimate that the concentration of CYP11A1 in glial cells is less than 4 pg per microgram total protein. This indicates that CYP11A1 can only be detected in human glial cells with more sensitive methods. To elucidate whether such low amounts of CYP11A1 could be responsible for the pregnenolone production in human glial cells, we investigated whether an alternative pathway may be involved in pregnenolone synthesis in glial cells.

Using the two CYP11A1 inhibitors AMG and KC, we found that pregnenolone production in the glial cells were not dose-dependently inhibited by CYP11A1 inhibitors, unlike adrenal H295R-S1 and MA-10 Leydig cells. Only when we overexpressed CYP11A1 in glial cells did we see a significant inhibitory effect by AMG on pregnenolone synthesis, which indicates that endogenous pregnenolone production by glial cells is independent of CYP11A1. In MA-10 and H295R-S1, progesterone and pregnenolone production were highest when cells were stimulated with 22(R)-HC, followed by 20α-HC, whereas the stereoisomer 22(S)-HC had no effect as expected (60, 61). This is in line with the side chain cleavage reaction by CYP11A1 where hydroxycholesterols with closest resemblance to the intermediates of the reaction are better metabolized to pregnenolone. However, in glial cells, 22(S)-HC is metabolized to pregnenolone at equal or higher rates than 22(R)-HC, suggesting that unlike adrenal and gonadal cells in the testis, there is no stereospecificity in the glial desmolase activity. Furthermore, 20α-HC appears to stimulate pregnenolone production more than the other two hydroxycholesterols in glial cells. This suggests that an alternative pathway to the CYP11A1 side chain cleavage reaction may be used to metabolize hydroxycholesterols in glial cells as compared to peripheral steroidogenic cells.

Previous studies in MGM-1 on steroidogenesis showed that DHEA synthesis by these cells can be dose-dependently induced by FeSO_4_ above 0.3 mM (37). The same trend was also observed in MGM-3 cells but the dose-dependent increase was not as clear. Although we hypothesized that pregnenolone synthesis could be increased by FeSO_4_ as well, our results from the present study show that FeSO_4_ had no effect on pregnenolone production in both MGM-1 and MGM-3 cells. The previous study also showed that FeSO_4_ dose-dependently increased cellular ROS in MGM-1 cells up to 3 mM. Here, we observed that FeSO_4_ only significantly increased cellular ROS at 500 μM in MGM-1 cells, with no significant increase at higher concentrations (data not shown). Nevertheless, in combination with our H_2_O_2_ results, our data suggests that pregnenolone synthesis by human glial cells is not dependent on ROS unlike DHEA synthesis. These results are in contrast to studies in C6 rat glial cells, where 10 mM H_2_O_2_ and FeSO_4_ both increased pregnenolone production by at least twofold (45). This difference could be due to species variations, as treatments of these two oxidants above 2 mM caused significant toxicity in MGM-1 and MGM-3 cells. Consequently, we have excluded these high concentrations from our analyses.

Our study highlights the discrepancies between the amount of pregnenolone produced by brain cells and the amount of CYP11A1 that is found in the brain. It has been reported that mitochondria from rat brains can convert radiolabeled cholesterol to pregnenolone at a rate of approximately 2.5 pmol/mg protein/hour while adrenal mitochondria have a conversion rate of approximately 15 pmol/mg/protein/hour (62). Even though the rate of CYP11A1 activity in the brain is only about six times lower than that of the adrenals, the mRNA and protein levels of CYP11A1 in the brain are more than 100 times lower (29, 32, 52). We question whether this small amount of CYP11A1 can account for the levels of pregnenolone found in human brains range, which range from 5.7 ng/g to 127.44 ng/g tissue with variations seen between different brain regions and between nondemented and Alzheimer’s disease patients (3, 4, 5, 63). Together with our data, we propose that an alternative pathway to CYP11A1 contributes to a major portion of the pregnenolone production in the brain.

This is not the first time an alternative pathway for neurosteroid synthesis has been proposed. Early experiments have shown that more pregnenolone and DHEA can be isolated from whole brain extracts when treated with FeSO_4_, FeCl_3_, lead tetraacetate, imidazole, or triethylamine (64). This idea was discussed by Lieberman *et al*., who suggested that the intermediates for pregnenolone synthesis found *in vivo* may be unstable oxygen radical products rather than stable intermediates that were isolated in *in vitro* experiments (65, 66, 67). The broad substrate specificity of CYP450 enzymes and the possibility that steroid synthesis can occur in multienzyme complexes pose uncertainties to the generally accepted pathway. Although there is not enough experimental evidence to support the potential reaction pathways proposed by Lieberman *et al*., their analyses indicate that the generally accepted steroidogenesis pathway is not without assumptions either.

Based on this, we recently proposed three possibilities for a hypothesized alternative pathway for CYP11A1-based steroid synthesis in the brain (33). Our data here provided evidence against both our first possibility that the alternative pathway is dependent on ROS and our second possibility that the second CYP11A1 isoform is responsible for pregnenolone production. To our knowledge, our study is the first to characterize CYP11A1b. Our qRT-PCR results suggest that exon 1 of the mRNA transcript for CYP11A1b may be spliced early, resulting in low CYP11A1b exon 1 expression but a total CYP11A1 expression higher than the sum of both isoforms. This may also indicate that CYP11A1b contributes to a non-negligible amount of total CYP11A1 expression in the brain, unlike in the periphery where CYP11A1a is clearly the dominant isoform. Given that the brain is rich in cholesterol and that all of CYP11A1b′s amino acid residues are found in CYP11A1a, we speculate that CYP11A1b could have a role in cholesterol homeostasis distinct to steroidogenesis, but more studies will be needed determine its exact function. We also observed that CYP11A1b is not strictly localized to the mitochondria unlike CYP11A1a, which is in line with current knowledge that CYP11A1b lacks the N-terminal mitochondrial-signaling peptide. However, we were unable to quantify CYP11A1b protein levels in the human brain and in glial cells due to the lack of unique amino acid peptides in CYP11A1b, lack of specific antibodies that could detect total CYP11A1 protein, and lack of a detectable signal in immunoblots using antibodies specific to CYP11A1a. Nevertheless, transfection of glial cells with CYP11A1b expression vectors did not alter pregnenolone synthesis while overexpression of the classical CYP11A1a significantly increased pregnenolone production. Therefore, CYP11A1b does not appear to be involved in pregnenolone synthesis.

Our results did support the third possibility that another CYP450 enzyme with desmolase activity in the brain can be used to synthesize pregnenolone. Although CYP46A1 could be a candidate given its high expression in brain, involvement in cholesterol metabolism, and structural similarity to CYP11A1, KC inhibits CYP46A1 activity potentially more effectively than CYP11A1 activity (68). Our data showed that the CYP450(s) responsible for producing pregnenolone is(are) not inhibited by AMG and KC. Furthermore, our preliminary studies indicated that CYP46A1 expression in glial cells is lower than that of CYP11A1 (data not shown), thus removing CYP46A1 as a likely candidate. Given that knockdown of FDXR resulted in decreased pregnenolone production while knockdown of POR did not, our data point toward a mitochondrial CYP450, of which there are seven known enzymes in humans: CYP11A1, CYP11B1, CYP11B2, CYP24A1, CYP27A1, CYP27B1, and CYP27C1 (27). Our qRT-PCR data showed that enzymes in the CYP11 family have low expression in the brain; therefore, CYP24A1 and CYP27 family enzymes are more probable candidates. Although we are currently investigating these enzymes to determine which of them are responsible for producing pregnenolone in the brain, we cannot eliminate the possibility that an undiscovered isoform of mitochondrial CYP450 may be involved in synthesizing pregnenolone. Furthermore, CYP1A1, CYP2E1, CYP2D7, CY1B1, and CYP2U1 have also been reported to have some mitochondrial localization, despite being found in the cytoplasm and/or endoplasmic reticulum as well (69), which adds to the list of potential CYP450 candidates. Thus, more extensive investigations into each of the mitochondrial CYP450s would be needed to elucidate the alternative pathway for pregnenolone synthesis in the human brain.

In sum, our study demonstrates that the human brain and human glial cells express very low levels of CYP11A1. Our data provides evidence for a CYP11A1-independent pathway for pregnenolone synthesis that is also independent of ROS, and we propose that another CYP450 other than CYP11A1 is responsible for synthesizing pregnenolone in the human brain. This suggests that it may be possible to specifically modulate CNS steroid synthesis pathways. Given that adverse effects can arise from systemic administration of steroid-modulating drugs and local administration of steroids is invasive, the alternative pathway for pregnenolone synthesis may be a promising investigative direction for CNS-specific neurosteroid-modulating therapies.

## Experimental procedures

### Cell culture

The human glioma cell lines MGM-1 and MGM-3 were a gift from Dr Hiroaki Kataoka (University of Miyazaki). MGM-1 and MGM-3 cells were grown in Dulbecco’s modified Eagle medium (DMEM; Gibco, Thermo Fischer Scientific, #11965092) with 10% heat-inactivated fetal bovine serum (FBS, Sigma; #12306C) plus 100 IU/ml penicillin and 100 μg/ml streptomycin (Gibco, Thermo Fischer Scientific; #15140122) at 37 °C and 5% CO_2_. The microglia cell line HMC3 (#CRL-3304) and the adrenal cortical carcinoma cell line NCI-H295R (referred to as H295R-S1; #CRL-2128) were purchased from American Type Culture Collection. HMC3 cells were grown in DMEM/F-12, GlutaMAX (Gibco, Thermo Fischer Scientific; #10565042) with 10% FBS, 100 IU/ml penicillin, and 100 μg/ml streptomycin at 37 °C and 5% CO_2_. H295R-S1 cells were grown in DMEM/F-12, GlutaMAX with 2.5% Nu-Serum (Corning; #355100), 100 IU/ml penicillin, 100 μg/ml streptomycin, plus ITS+ Premix Universal Culture Supplement at a concentration recommended by the manufacturer (Corning; #354352). NHAs were purchased from Lonza (#CC-2565). NHA cells were grown at 37 °C and 5% CO_2_ in astrocyte growth medium BulletKit (Lonza; #CC-3186): Astrocyte basal medium plus rhEGF, insulin, ascorbic acid, GA-1000, L-glutamine, and 3% FBS. MA-10 cells were a gift from Dr Mario Ascoli (University of Iowa). MA-10 cells were grown in DMEM/F12, GlutaMAX with 5% FBS, 2.5% horse serum (Gibco, Thermo Fischer Scientific; #26050088), 100 IU/ml penicillin, and 100 μg/ml streptomycin at 37 °C and 3.5% CO_2_. NHA cells were passaged using ReagentPack Subculture Reagents (Lonza; #CC-5034), and all other cell lines were passaged using trypsin/EDTA (Gibco, Thermo Fischer Scientific; #25200056). A maximum of 10 passages were used for all cell lines.

### Cell treatments for steroid measurements

FGIN-1-27 (#18461) and 22(S)-HC (#21399) were purchased from Cayman Chemicals. Other compounds used to treat cells were purchased from Sigma: XBD173 (#SML1223), AMG (#A9657), KC (#K1003), 22(R)-HC (#H9384), 20α-HC (#H6378), Trolox (#238813), hydrogen peroxide solution (#216763), iron (II) sulfate (#F8633), SNAP (#N3398), deferoxamine mesylate (#1166003), 3-Hydroxy-1,2-dimethyl-4(1H)-pyridone (deferiprone; #379409), and diphenyleneiodonium chloride (#D2926).

For steroid synthesis experiments, cells were seeded in 24- or 96-well plates and grown to 70% to 80% confluency. Before treatment, cells were washed three times with PBS to remove steroids from the serum in complete media. Drugs used for treating cells were dissolved in dimethyl sulfoxide, PBS, or ethanol depending on solubility to make stock solutions with at least 200X concentration. Stock solutions were diluted in serum-free base media to treatment concentrations immediately before treatment. Appropriate solvent controls were made based on concentration of solvent in the highest concentration treatment condition. Cells were incubated with treatment media for 2 h, after which the media was collected and stored at 80 °C until used for steroid measurement. Cells were then lysed with 0.1 M NaOH solution, and protein quantity was measured using the Bradford assay (VWR; #97065-020) to normalize steroid measurements.

The levels of major steroids produced by cells were measured by performing ELISA on collected media. Pregnenolone was measured in media from MGM-1, MGM-3, NHA, HMC3, and H295R-S1 cells using pregnenolone ELISA kits (Abnova; #KA1912). Progesterone was measured in media from MA-10 cells using progesterone ELISA kits (Cayman Chemical; #582601). ELISA assays were performed according to protocols provided by manufacturers.

### CYP11A1 isoforms overexpression

MGM-1 cells were seeded into 24-well plates at a density of 50,000 cells/well. The cells were then transfected with 1 μg of pCMV-entry mammalian expression vector (Origene; #PS100001), CYP11A1 transcript variant 1 (Myc-DDK-tagged; CYP11A1a) expression vector (Origene; #RC207121), or CYP11A1 transcript variant 2 (Myc-DDK-tagged, CYP11A1b) expression vector (Origene; #RC211728) using Lipofectamine 3000 (Thermo Fischer Scientific; #L3000001), and Opti-MEM (Gibco, Thermo Fischer Scientific; #31985062), according to manufacturer’s instructions. After 24 h, culture media was replaced. Transfected cells were then selected with 1 mg/ml geneticin (Gibco, Thermo Fischer Scientific; #10131027) for 7 days. qRT-PCR, immunofluorescence, and Western blot were used to confirm CYP11A1a or CYP11A1b expression. Steroid measurements for transfected cells were performed as described previously.

### siRNA knockdown of FDXR and POR

MGM-1 cells were seeded into 12-well plates at a density of 60,000 cells/well or into 6-well plates at a density of 120,000 cells/well. The cells were transfected with 5 nM scrambled negative control siRNA duplexes or gene-specific siRNA duplexes (Origene; #SR303637 for *POR* and #SR301560 for *FDXR*) using siTran 2.0 transfection reagents (Origene; #TT320001), according to manufacturer’s instructions. Culture media was replaced 18 h after transfection. Transfected cells in 12-well plates were collected 48 h after transfection for RNA analysis by qRT-PCR. After 72 h post-transfection, transfected cells in 6-well plates were washed and treated with serum-free media for 2 h, after which media was collected for steroid measurement by ELISA and the cell pellet collected for protein quantification and Western blotting. Knockdown efficiency was determined by both qRT-PCR and immunoblot analyses.

### RNA extraction and qRT-PCR

Total RNA was extracted from cell pellets consisting of 10^6^ cells and DNAse treatment was performed to remove genomic DNA using the RNAqueous-Micro Kit (Thermo Fischer Scientific; #AM1931). Human CNS tissue RNA for total brain (#636530), cerebellum (#636535), cerebral cortex (#636561), parietal lobe (#636571), occipital pole (#636570), temporal lobe (#636564), and spinal cord (#636554) were purchased from Takara Bio. RNA (1000 ng) were reverse transcribed into complementary DNA using PrimeScript RT Master Mix (Takara; #RR036B). qRT-PCR was then performed in 384-well plates using SYBR Select Master Mix (Thermo Fischer Scientific; #4472908) with 100 nM forward and reverse primers (Integrated DNA Technologies). Primers were designed using the NCBI Primer Blast tool, where primers pairs that spanned exon–exon junctions were preferentially selected. Plates were assayed on a CFX384 Touch Real-Time PCR Detection System (Bio-Rad). Sequences of all forward and reverse primers are listed in Table S2. For each gene, 20 ng complementary DNA was used for detection. Data were analyzed using Bio-Rad CFX Maestro software. Relative quantification analysis was performed using the 2^-ΔCT^ method except when comparing transfected cells to negative controls, where the 2^-ΔΔCT^ method was used instead. *CYP11A1* expression was detected by the *CYP11A1* exon 3 to 4 primers listed in Table S2, unless a particular variant was specified.

### Cell lysate preparation and Western blotting

Cell pellets consisting of 2 × 10^6^ cells were lysed in radioimmunoprecipitation assay (RIPA) buffer with 2% protease inhibitor (Thermo Fischer Scientific; #A32955). Fifteen migrograms of total proteins were resolved on 4% to 20% precast polyacrylamide gels (Bio-Rad; #4561096) and transferred to polyvinylidene difluoride membranes (Sigma; #ISEQ00010). After transfer, membranes were blocked for 30 min with blocking solution consisting of 5% bovine serum albumin (Equitech-Bio; #BAH65) dissolved in PBS with Tween-20 (PBST). Membranes were then incubated with specific primary antibodies diluted in blocking solution overnight at 4 °C, washed three times with PBST, incubated with corresponding secondary antibodies for 1 h at room temperature (RT), and washed three times with PBST. Antibodies were detected using Clarity Western ECL Substrate system (BioRad; #1705061), which allows detection of signals for >0.3 ng protein recognized by antibodies, and visualized using a Western blot imaging system (Azure Biosystems; c600). The following primary antibodies were used: anti-CYP11A1 rabbit mAb (Cell Signaling Technology; #14217, 1:1000 dilution, antigen: human CYP11A1 N terminus), anti-CYP11A1 rabbit pAb (Proteintech; #13363-1-AP, 1:1000 dilution, antigen: CYP11A1 aa 1–300), anti-CYP11A1 rabbit pAb (Abcam; #ab75497, 1:500 dilution, antigen: human CYP11A1 aa 288–337), anti-CYP11A1 rabbit pAb (Abcam; #ab232763, 1:1000 dilution, antigen: CYP11A1 aa 350–521), anti-GAPDH rabbit mAb (Cell Signaling Technology; #2118, 1:2000 dilution), anti-FDXR rabbit pAb (Proteintech; #15584-1-AP, 1:1000 dilution), anti-ADX rabbit mAb (Abcam; #ab108257, 1:1000 dilution), antibeta actin mouse mAb (Abcam; #ab8226, 1:3000 dilution), anti-CYP reductase rabbit pAb (Abcam; #ab13513, 1:1000 dilution), and anti-Myc-tag rabbit mAb (Cell Signaling Technology; #2278S, 1:1000 dilution).

### ROS measurements

Changes in ROS levels were measured using a Cellular ROS Assay Kit (Abcam; #ab186027), performed according to manufacturer’s instructions. Briefly, cells were seeded in 96-well plates at 70% to 80% confluency and incubated with ROS Red Stain working solution provided in the kit for 1 h prior to treatment. Treatment solutions made at 10× concentration were then added to the wells to achieve the appropriate treatment concentrations. After a 2 h incubation, fluorescence was measured at 520 nm/605 nm using a Biotek Synergy H1 Hybrid Multi-Mode Microplate Reader. ROS measurements were normalized to that of the control condition.

### Immunocytochemistry

Cells were seeded onto glass coverslips in 12-well plates and grown to 50% confluency. For immunocytochemistry, cells were incubated with 250 nM MitoTracker Red CMXRos (Thermo Fischer Scientific; #M7512) in complete media for 40 min. Then, cells were fixed in 4% paraformaldehyde for 10 min and permeabilized with 0.1% Triton X for 10 min, with three PBS washes between each step. Blocking was then performed for 30 min with 5% donkey serum (Sigma; #D9663) + 0.5% bovine serum albumin (Equitech-Bio; #BAH65). Cells were incubated overnight at 4 °C with anti-CYP11A1 rabbit pAb (Proteintech; #13363-1-AP, 1:400 dilution in PBS) or anti-Myc-tag rabbit mAb (Cell Signaling; #2278S, 1:200 dilution). Following primary antibody incubation, cells were washed three times with PBS and incubated with Alexa Fluor 488 goat anti-rabbit IgG (H + L) secondary antibody (Thermo Fischer Scientific; #A11008, 1:1000 dilution in PBS) for 1 h at RT. After three PBS washes, the coverslips were mounted onto microscope slides with Vectashield Vibrance mounting medium with 4′,6-diamidino-2-phenylindole (Vector Laboratories; #H-1800). The slides were then imaged at 63× magnification using Zeiss LSM 880 with Airyscan Confocal Microscope.

### RNAscope *in situ* hybridization

Formalin-fixed paraffin-embedded cerebellum and cerebral cortex tissue slices from a 63-year-old male, as well as cerebral cortex tissue slices from a 92-year-old female, were obtained from ACD Bio. Both donors were free from any neurological disorders. Two probes were used for the duplex ISH assay: one custom probe targeting exon 2 to 8 of *CYP11A1* and a second probe targeting *MBP* from the ACD Bio catalog (#411058). The ISH assay was performed, and images were taken by ACD Bio according to previously published procedures (38).

### Shotgun proteomics MS

H295R-S1, MGM-1, and NHA cells were pelleted by centrifuging at 200*g* after three PBS washes. Pellets consisted of 5 × 10^6^ cells and were lysed using RIPA buffer with 2% protease inhibitor (Thermo Fischer Scientific; #A32955). Eighty micrograms of total proteins were resolved on 12% acrylamide gels (Bio-Rad; #1610185). Protein bands were visualized by staining with Coomassie blue (Bio-Rad; #1610786) for 1 h. Bands at 40 kDa, 50 kDa, and 60 kDa were excised and shipped to Creative Proteomics for shotgun proteomics. A summary of the protocol was provided by Creative Proteomics. Briefly, the protein bands were digested with trypsin and resuspended in 0.1% formic acid before LC-MS/MS analysis performed on a Nanoflow UPLC system. A full scan was performed between 300 to 1650 m/z at resolution of 60,000 at 200 m/z. Raw MS files were analyzed and searched against human protein database using Maxquant 1.6.2.6 (Max-Planck Institute of Biochemistry) to obtain a list of matching proteins in each band.

### MS for pregnenolone detection

Steroids in the media were extracted twice with 1-chlorobutane (Sigma; #34958) using media:solvent ratio of 1:2. The top organic layer was collected and dried using a rotary evaporator. Pregnenolone was derivatized with 1-amino-4-methylpiperazine (AMP; Sigma; 255688) to increase detection sensitivity based on a published method (70). Briefly, 400 μl of 2 mg/ml AMP in methanol and 400 μl of 1% acetic acid in methanol were added to dried samples and vortexed for 10 min before evaporation under a stream of nitrogen for 35 min. Dried, derivatized samples were reconstituted in 200 μl of 50% methanol + 0.1% formic acid solution, where 100 μl of sample solution was transferred to LC-MS vials. Cell pellets were lysed in RIPA buffer with 2% protease inhibitor (Thermo Fischer Scientific; #A32955), and protein concentration was quantified using the bicinchoninic acid assay to use for normalization (Thermo Fischer Scientific; #23225).

To generate a calibration curve, 400 μl of 1 μg/ml pregnenolone standard (Sigma; #P9129) were dried, derivatized as described previously, and reconstituted in 200 μl 50% methanol for a final concentration of 2000 ng/ml pregnenolone-AMP. An internal standard (IS) stock solution was prepared in the same way using d4-pregnenolone (Sigma; #809845). Final concentrations for the calibration curve were prepared at 100, 50, 20, 10, 5, 2, 1, 0.5, and 0.25 ng/ml pregnenolone with 50 ng/ml IS in 50% methanol + 0.1% formic acid using serial dilutions.

The LC-MS/MS measurements were performed with an Agilent Poroshell 120 EC-C18 column (2.7 μm, 2.1 × 150 mm) using Agilent Infinity II HPLC connected to a QTRAP 6500+ mass spectrometer (AB Sciex). Mobile phase A was 0.1% formic acid in H_2_O, while mobile phase B was 0.1% formic acid in acetonitrile. MS detection was performed in positive mode. Multiple reaction monitoring transitions used were as follows: precursor ion (*m/z* = 414) → product ion (*m/z* = 99) for pregnenolone-AMP and precursor ion (*m/z* = 418) → product ion (*m/z* = 99) for IS. MS parameters were optimized to achieve optimal sensitivity: ion spray voltage was 4500 V, source temperature was 300 °C, curtain gas/ion gas 1/ion gas 2 were 20 psi, declustering potential was 79.5 V, collision energy was 26.3, and dwell time was 50 ms. The data were acquired and analyzed using Analyst 1.7 (SCIEX) software.

### MS for detection of multiple steroids

H295R-S1 and MGM-1 cells were treated for 2 h with 76 mM AMG or 100 μM KC with or without 50 μM 22(R)-HC stimulation in serum-free media. One milliliter of supernatant was collected for each sample and lyophilized using FreeZone 1 l -50C Freeze Dryers (Labconco; #7740021). Lyophilized samples were extracted with 200 μl of methyl tert-butyl ether three times and the combined organic layers were dried using a Speed Vac. Samples were reconstituted in 200 μl of 10 + 90 (v/v) mixture of dH_2_O:ethanol with 0.1% formic acid (aqueous) and transferred to HPLC vials. LC-MS/MS was performed by the Proteomics Service, Research Institute of McGill University Health Centre (RIMUHC) on an AB Sciex 5600 Time of Flight Mass Spectrometer connected to a Nexera XR LC-20 ADXR UHPLC system using a Zorbax (Agilent) Eclipse C18 (2.1 × 50 mm, 1.8 μm) column and autosampler. Mobile phase A was 0.1% formic acid in water, and mobile phase B was 0.1% formic acid in acetonitrile. MS parameters were as followed: ion spray voltage was 5500 V, source temperature was 500 °C, curtain gas 30 L/min, ion source gas 1 and 2 were 50 l/min, collision energy was 10, and declustering potential was 100 V. Product ions scanned were as follows: androstenedione (*m/z* = 287.2), testosterone (*m/z* = 289.2), DHEA (*m/z* = 289.2), progesterone (*m/z* = 315.2), and pregnenolone ([M-18]+; *m/z* = 229.2). Analytical standards in solution were used to establish linearity and dynamic range. Data acquisition and analysis were done using MultiQuant 3.0.2 and Analyst TF 1.7 (AB Sciex).

### Statistics

Statistical analyses of steroid and ROS measurements were performed using GraphPad Prism 9.2.0 (GraphPad Software). Statistical significance was determined using one-way ANOVA followed by Dunnett’s multiple comparison test. For multiple comparison tests, each value was compared to the no-drug solvent control group unless otherwise indicated.

## Data availability

All data are contained within the article and/or available upon request.

## Supporting information

This article contains supporting information.

## Conflicts of interest

The authors declare that they have no conflicts of interest with the contents of this article.

## Supporting information

CYP11A1-Independent Pregn Support Tables & Figures JBC3

CYP11A1-Independent Pregn Proteomics-Dataset S1-JBC2
