# Supplementary material for: The neurosteroid pregnenolone is synthesized by a mitochondrial P450 enzyme other than CYP11A1 in human glial cells
Source: J Biol Chem. 2022 Jun 7;298(7):102110. doi: 10.1016/j.jbc.2022.102110 (PMC9278081; doi:10.1016/j.jbc.2022.102110)
Supplement: CYP11A1-Independent Pregn Support Tables & Figures JBC3 [file mmc1.pdf]

Supplementary Information

The neurosteroid pregnenolone is synthesized by a mitochondrial P450 enzyme other than CYP11A1 in human glial cells

Yiqi Christina Lin[1], Garett Cheung[1], Edith Porter[2], Vassilios Papadopoulos[1,*]

[1]Department of Pharmacology and Pharmaceutical Sciences, School of Pharmacy, University of Southern California, Los Angeles, CA; [2]Department of Biological Sciences, California State University, Los Angeles, CA

Tables S1-S2

Figures S1-S10

Table S1: List of Cell Lines Used

Cell Line	Species	Phenotype	Source
MA-10	Mouse	Leydig cell	Leydig cell tumor
H295R-S1	Human	Adrenocortical cell	Adrenocortical carcinoma
MGM-1	Human	Oligodendrocyte (MBP+)	Glioblastoma
MGM-3	Human	Oligodendrocyte (MBP+)	Glioblastoma
NHA	Human	Astrocyte (GFAP+)	Primary cells
HMC3	Human	Microglia (Iba1+)	Immortalized primary cells

MBP: myelin basic protein; GFAP: glial fibrillary acidic protein; Iba1: allograft inflammatory factor 1

Table S2: Sequences of primers used for qRT-PCR

Gene	Forward Primer	Reverse Primer
CYP11A1 exon 3 – 4	GCTTTGCCTTTGAGTCCATCA	CTCGGGGTTCACTACTTCCTC
CYP11A1 variant 1 exon 1	CACGCTCAGTCCTGGTCAAA	GGGGATCTCATTGAAGGGGC
CYP11A1 variant 2 exon 1	ATTCCGAAATTGGGTCCGCC	GAGTCCGTGTTGGGGAACTT
FDX1	TTCAACCTGTCACCTCATCTTTG	TGCCAGATCGAGCATGTCATT
FDXR	CTGAGGCAGAGTCGAGTGAAG	CCCGAAGCTCCTTAATGGTGA
TSPO	GCCATACGCAGTAGTTGAGTG	CCTGCTCTACCCCTACCTGG
STAR	ACAGACTTCGGGAACATGC	TGAGTAGCCACGTAAGTTTGG
CYP17A1	GTTGTTGGACGCGATGTCTA	TTCGTATGGGCACCAAGACT
CYP19A1	TGGAAATGCTGAACCCGATAC	AATTCCCATGCAGTAGCCAGG
CYP21B	AAGGACAGGTCCGGGTAGTT	CCAAGAGGACCATTGAGGAA
CYP11B1	GAGGCCTGAGCGCTATAACC	TGGAGGTGTTTCAGCACATGG
CYP11B2	TTCAACCGCCCTCAACACTAC	GGAAACGCTGTCGTGTCCA
HSD3B1	CACATGGCCCGCTCCATAC	GTGCCGCCGTTTTTCAGATTC
HSD3B2	AGAACGGCCACGAAGAAGAG	TGGGTCTTAACGCACAAGTGT
HSD17B1	ACGTGAATGTAGTAGGGACTGT	GCGCAATAAACGTCATTGAAAGG
HSD17B2	AGCTGAGGAATTGCGAAGAAC	GCAACCTTGCTGTAAGCATCT
HSD17B3	CTGGCGAAGTGCGTGAGATT	GAGTACGCTTTCCCAATTCCAT
HSD17B4	TGAGGGATCGTTCCTTTGCTA	CGTGTCACTTGGAATGAACCC
AKR1C1	TAGGCAACTGTGTCATGGTGG	AAAGGCAGCGAAGGATTCAGA
AKR1C2	GTTGCCAGCTCATTGCTCTT	CCAGGACAGGCATGAAGTGA
AKR1C3	GTCATCCGTATTTCAACCGGAG	CCACCCATCGTTTGTCTCGTT
AKR1C4	AGGTGAGACGCCACTACCAA	GTTTGCTCTGGTTGAGGTAAGG
SRD5A1	TCAGACGAACTCAGTGTACGG	CGTAGTGGACGAGGAACATGG
SRD5A2	ACTGCTCAATCGAGGGAGG	CACCCAAGCTAAACCGTATGTC
STS	CCTACTGTTCTTTCTGTGGGAAG	CGAGGTCGTCAGCCATCAC
SULT2A1	CGTGATGAGTTCGTGATAAGGG	GGCAGAGAATCTCAGCCAACC
SULT2B1	GTTGCCAGGTGAATACTTCCG	CCCGCACATCTTGGGTGTT
HSD11B1	GCCTGCTTAGGAGGTTGTAG	CCTTGGAGCATCTCTGGTCT
HSD11B2	GGACCTGACCAAACCAGGAG	TTCACCTCCATGCAGCTACG
TUBA1	TCGATATTGAGCGTCCAACCT	CAAAGGCACGTTTGGCATACA
ACTB	CCTTGCACATGCCGGAG	GCACAGAGCCTCGCCTT
POR	TTTCGCTCATCGTGGGTCTC	CGATGATGTTCCTCCCCGTT

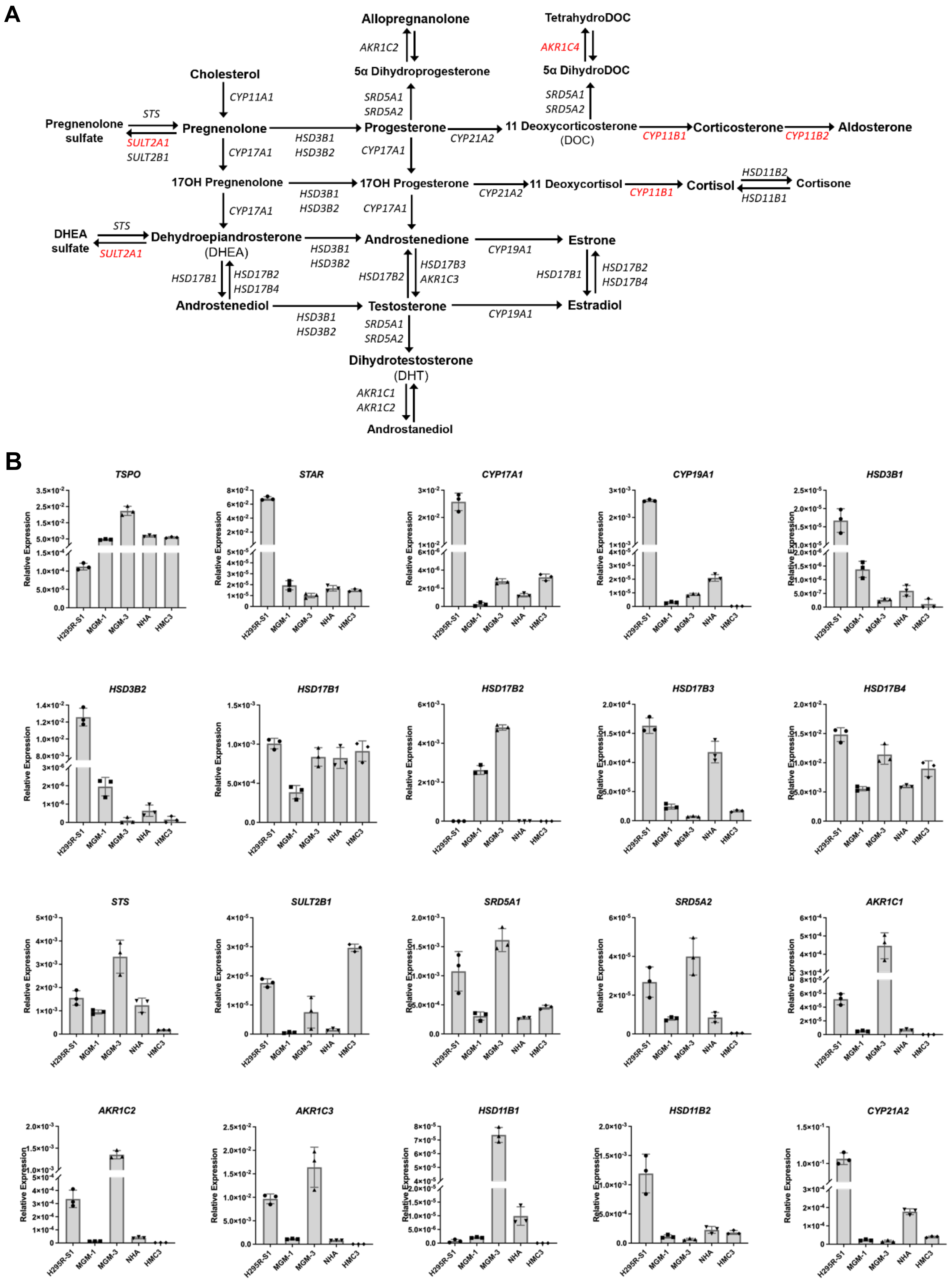
A
Allopregnanolone
AKR1C2
5α Dihydroprogesterone
TetrahydroDOC
AKR1C4
5α DihydroDOC
Cholesterol
CYP11A1
SRD5A1
SRD5A2
Pregnenolone sulfate
STS
SULT2A1
SULT2B1
Pregnenolone
HSD3B1
HSD3B2
Progesterone
CYP21A2
11 Deoxycorticosterone (DOC)
CYP11B1
Corticosterone
CYP11B2
Aldosterone
CYP17A1
17OH Pregnenolone
17OH Progesterone
11 Deoxycortisol
Cortisol
HSD11B2
HSD11B1
Cortisone
DHEA sulfate
Dehydroepiandrosterone (DHEA)
Androstenedione
CYP19A1
Estrone
HSD17B1
HSD17B2
HSD17B4
HSD17B3
AKR1C3
Androstenediol
Testosterone
Estradiol
Dihydrotestosterone (DHT)
AKR1C1
Androstanediol
B
TSPO
STAR
CYP17A1
CYP19A1
HSD3B1
HSD3B2
HSD17B1
HSD17B2
HSD17B3
HSD17B4
STS
SULT2B1
SRD5A1
SRD5A2
AKR1C1
AKR1C2
AKR1C3
HSD11B1
HSD11B2
CYP21A2
Relative Expression
H295R-S1
MGM-1
MGM-3
NHA
HMC3

Figure S1: mRNA expression of steroidogenesis machinery in H295R-S1 and human glial cells. (A) Schematic diagram of the steroidogenesis pathway with summary of steroidogenic enzyme expression in human glial cells. Steroids are written in bold while the gene names of steroidogenic enzymes responsible for producing the steroid are written in italics. Enzymes with detectable mRNA expression in MGM-1, MGM-3, NHA, or HMC3 cells are written in black, while enzymes without detectable expression in glial cells are written in red. The mRNA for all the enzymes could be detected in H295R-S1 cells. (B) qRT-PCR analyses of genes important for steroidogenesis in H295R-S1, MGM-1, MGM-3, NHA, and HMC3 cells. Gene expression is shown as relative expression to α-tubulin. Data are presented as mean ± SD, N=3. Each data point represents total RNA extracted from cells of a different passage for each cell line. Enzymes involved in the steroidogenesis pathway include cytochrome P450 side-chain cleavage (*CYP11A1*), cytochrome P450 17α-hydroxylase/C17,20-lyase (*CYP17A1*), aromatase (*CYP19A1*), 21-hydroxylase (*CYP21A2*), 11β-hydroxylase (*CYP11B1*), aldosterone synthase (*CYP11B2*), 3β-hydroxysteroid dehydrogenase (*HSD3B1*, *HSD3B2*), 17β-hydroxysteroid dehydrogenase (*HSD17B1, HSD17B2, HSD17B3, HSD17B4*), 11β-hydroxysteroid dehydrogenase (*HSD11B1, HSD11B2*), 5α-reductase (*SRD5A1, SRD5A2*), 3α-hydroxysteroid dehydrogenase (*AKR1C1, AKR1C2, AKR1C3, AKR1C4*), steroid sulfatase (*STS*), and sulfotransferase (*SULT2A1, SULT2B1*). Translocator protein (*TSPO*) and steroid acute regulatory protein (*STAR*) mediate cholesterol transport into the mitochondria, which is the rate-limiting step for classical steroid synthesis.

A. Cerebellum gray matter (63y male)

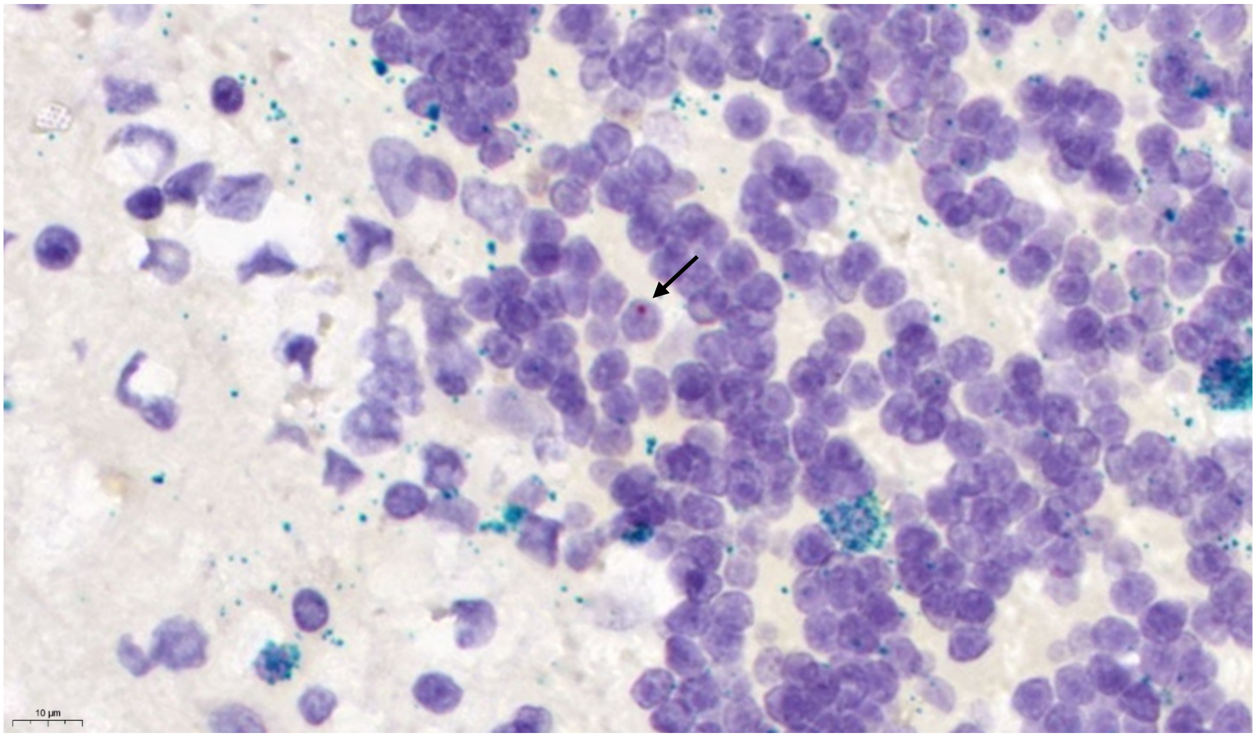

B. Cerebellum gray matter with Purkinje cells (63y male)

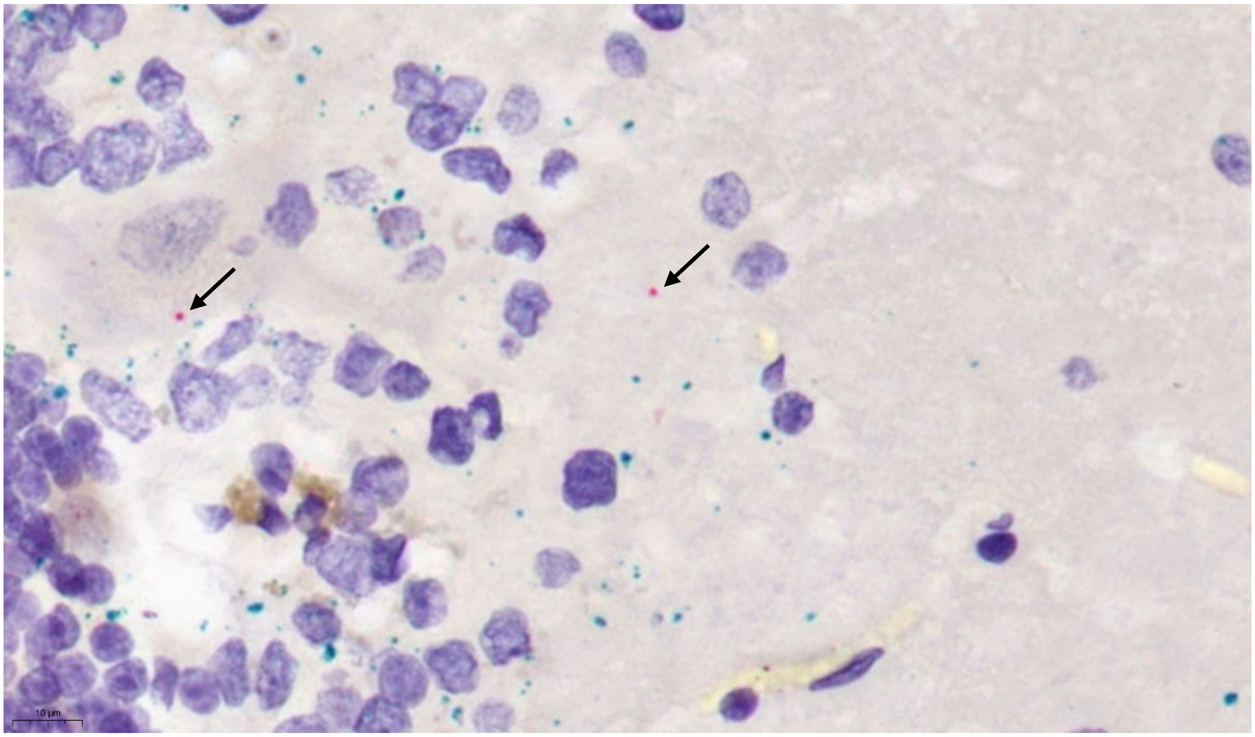

C. Cortex gray matter (63y male)

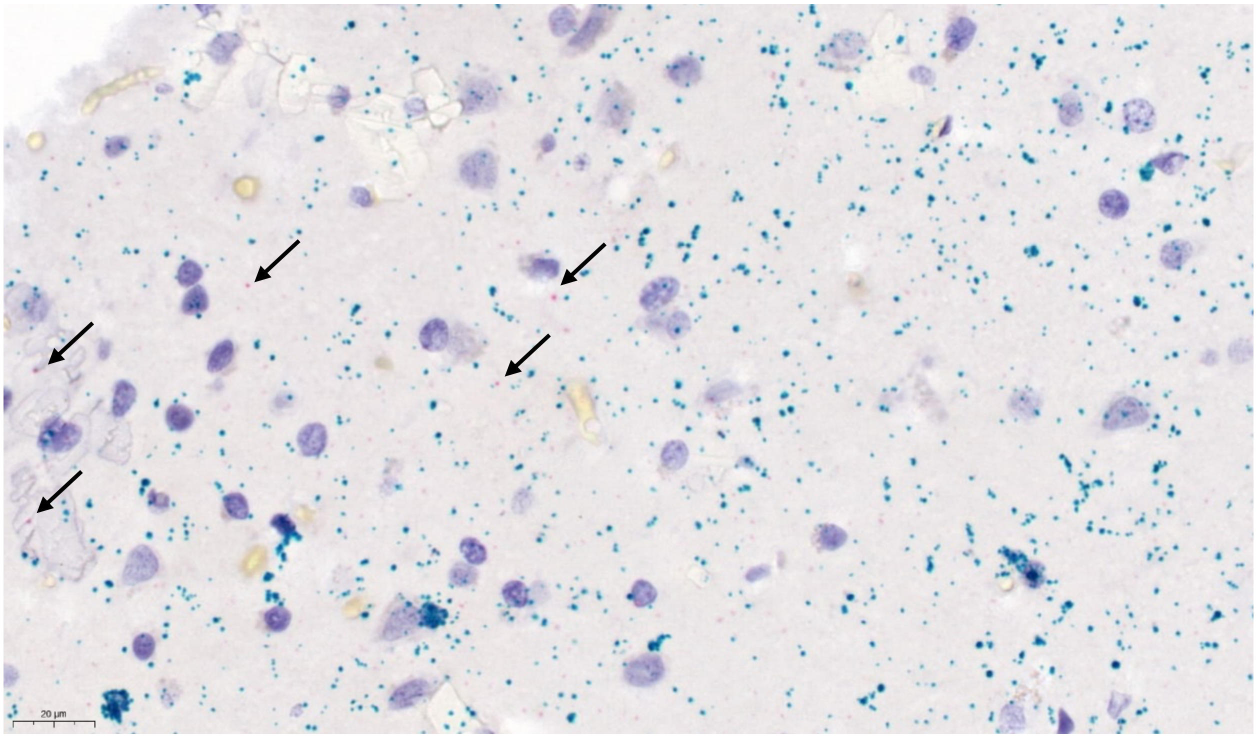

D. Cerebellum white matter (63y male)

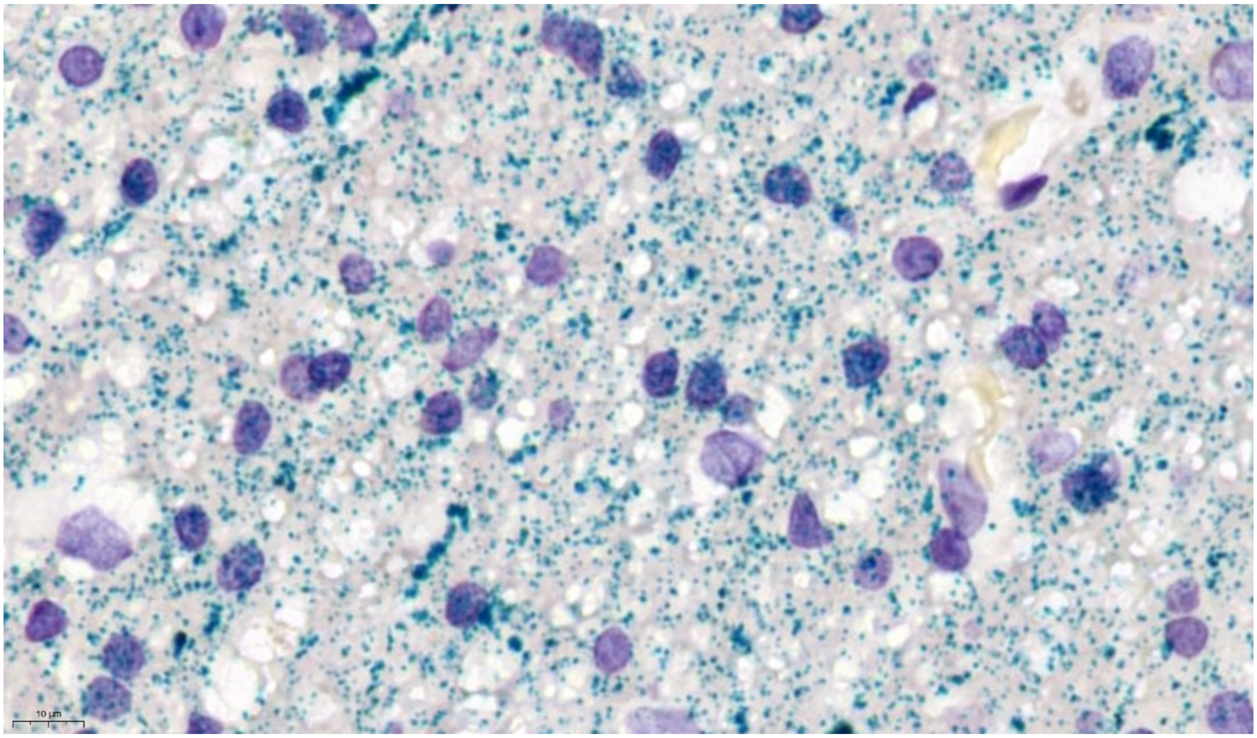

E. Cortex white matter (63y male)

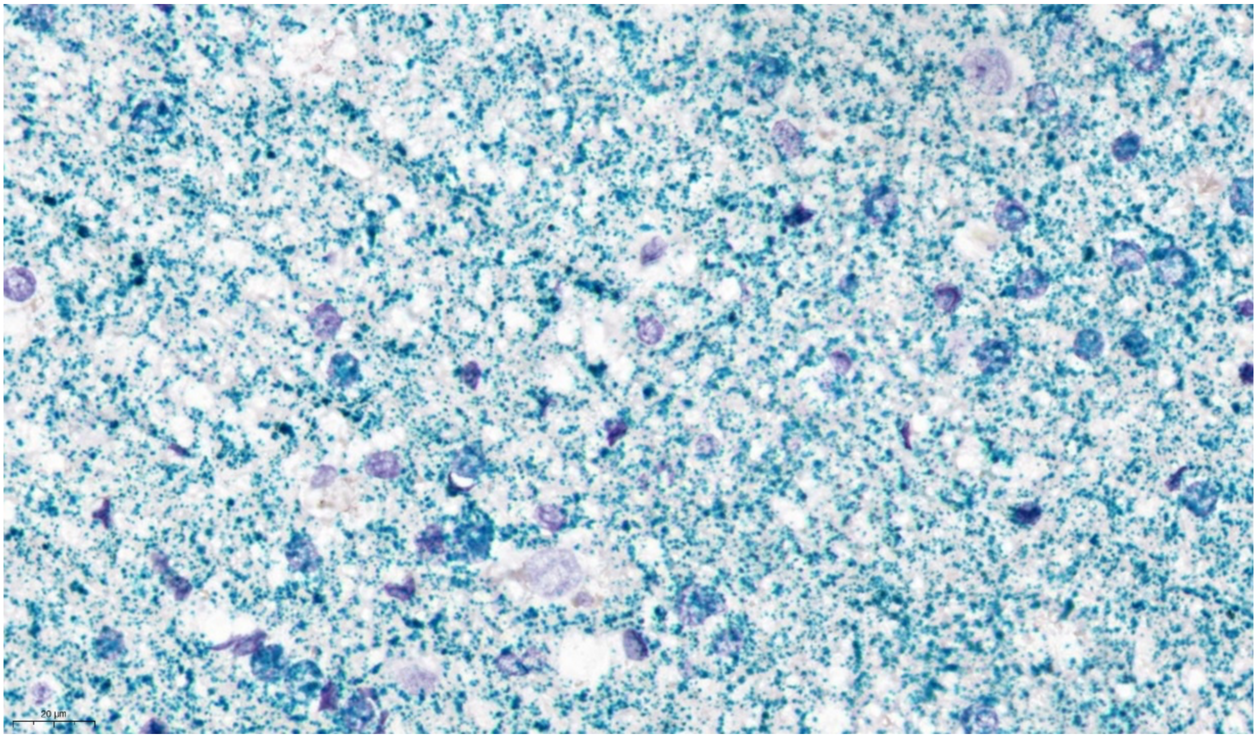

F. Cortex gray matter (92y female)

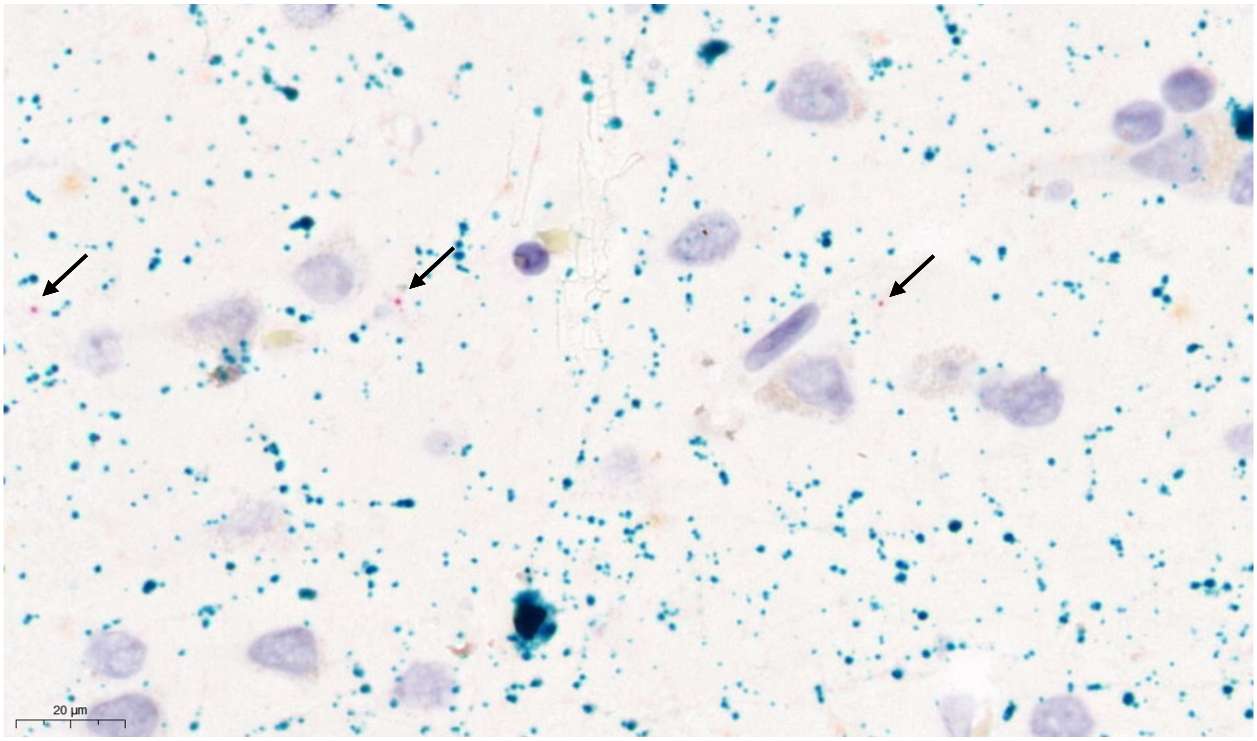

G. Cortex white matter (92y female)

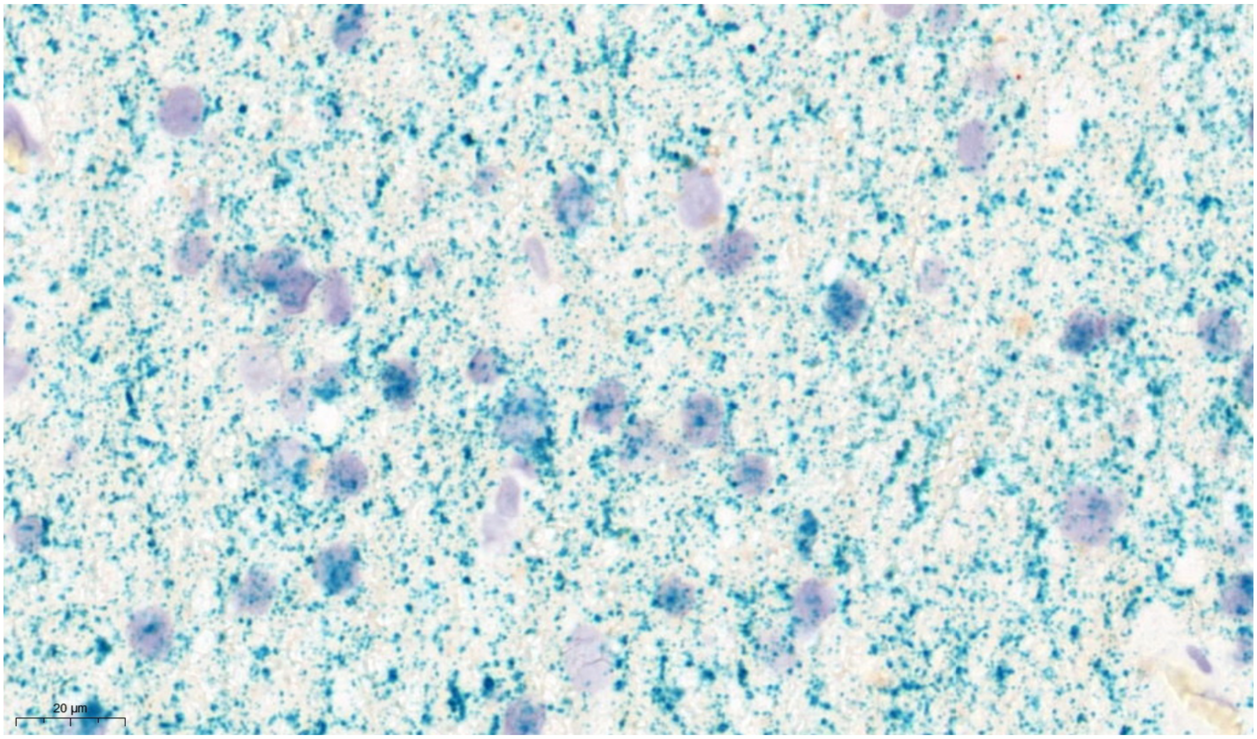

H. Cortex white matter (92y female)

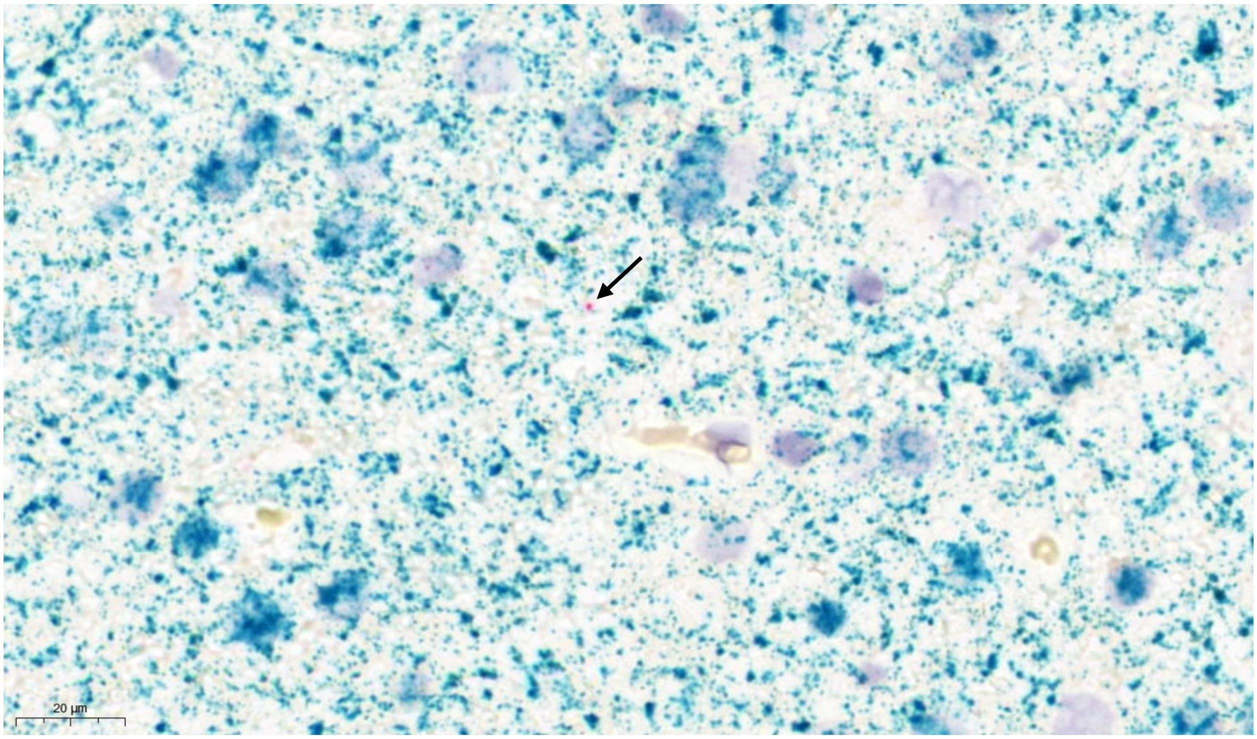

I. Positive control (63y male)

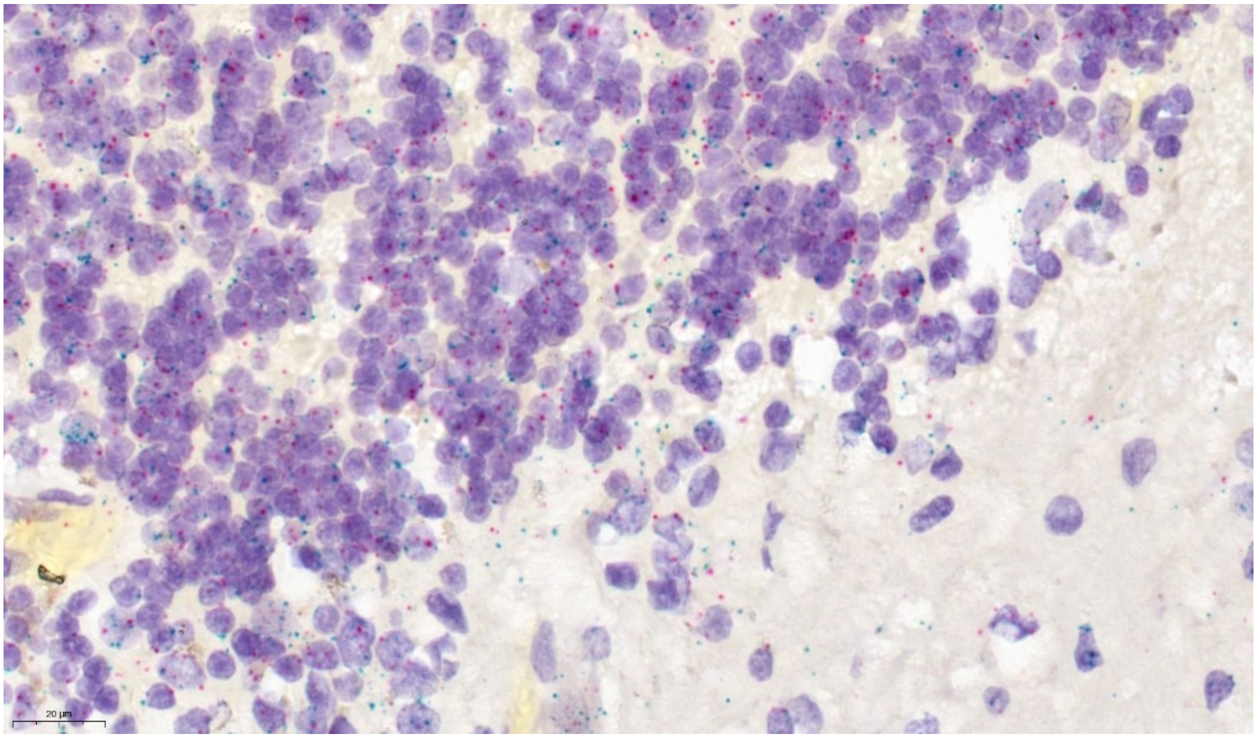

Figure S2: RNAscope *in situ* hybridization analyses of human brain tissue. A chromogenic assay was performed with two targets: CYP11A1 (red) and myelin basic protein (MBP; teal). Each punctate dot represents one molecule of mRNA. Tissues from two donors were used: cerebellum and cerebral cortex tissue from a 63y male and cerebral cortex tissue from a 92y female. (A – D) Full images of Figure 1D – G. CYP11A1-positive cells can be found in the gray matter of the cerebellum and cortex (A – C, F) but little to none in white matter (D, E, G, H), as indicated by black arrows. CYP11A1-positive cells were only observed in the cortex white matter of one donor (H). (I) Representative positive control for assay (red = RNA polymerase II subunit a (Polr2a), teal = peptidylprolyl isomerase B (PPIB))

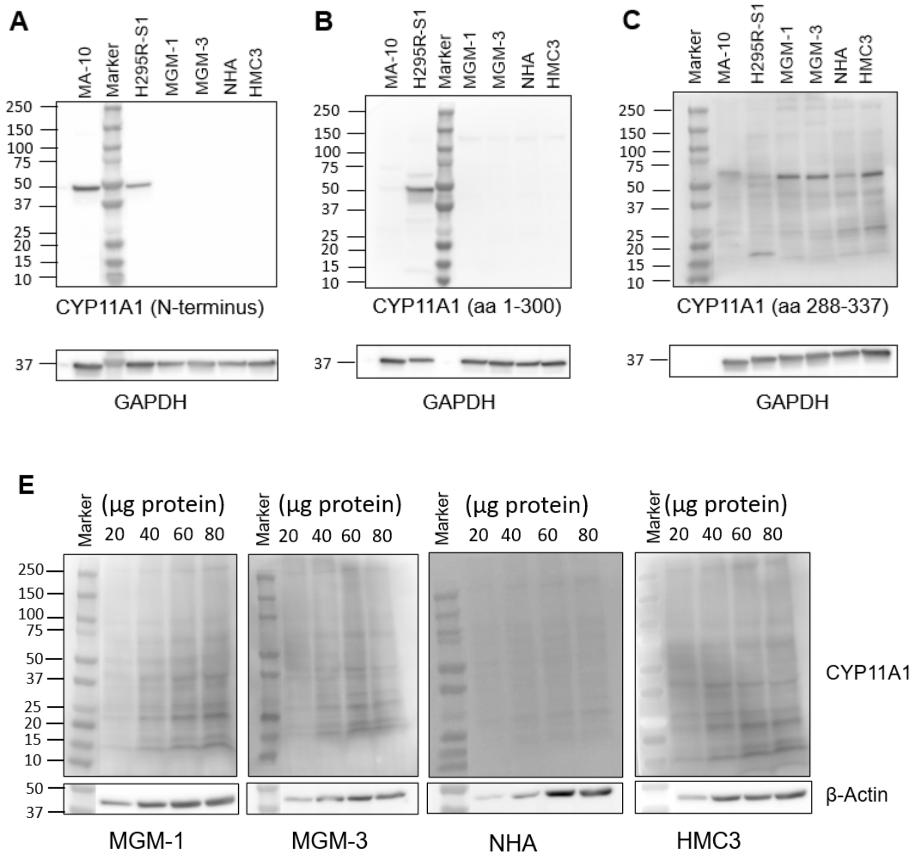

Figure S3: **Western blot analyses of CYP11A1 in human glial cells, with MA-10 and H295R-S1 as positive control**. (A) Full membrane image of immunoblot shown in Figure 2D. Anti-CYP11A1 antibody was raised against the N-terminus sequence of human CYP11A1. (B-D) Immunoblots using different anti-CYP11A1 antibodies. 15 µg of total cell lysate was loaded into each lane, with GAPDH as a loading control. Antigens used to raise each antibody are: (B) CYP11A1 aa 1– 300, (C) human CYP11A1 aa 288 – 337, (D) human CYP11A1 aa 350 – 521. GAPDH in (C) is the same as the GAPDH image in Figure 2E. The same membrane was blotted with FDXR, FDX1, and CYP11A1 (aa 288 - 337) antibodies one after the other with stripping in between. Results for FDXR and FDX1 can be found in Figure 2E. To confirm antibody specificity for blots (C) and (D), protein bands at 40, 50, and 60 kDa were excised and analyzed by shotgun proteomics; however, no peptide sequence corresponding to CYP11A1 could be found (see Dataset S1). (E) Western blot analyses of CYP11A1 in human glial cells using the same antibody as (A), with 20, 40, 60, or 80 µg of total cell lysate loaded into each lane. No specific bands for CYP11A1 can be observed for any of the glial cell lines. β-actin was used as a loading control.

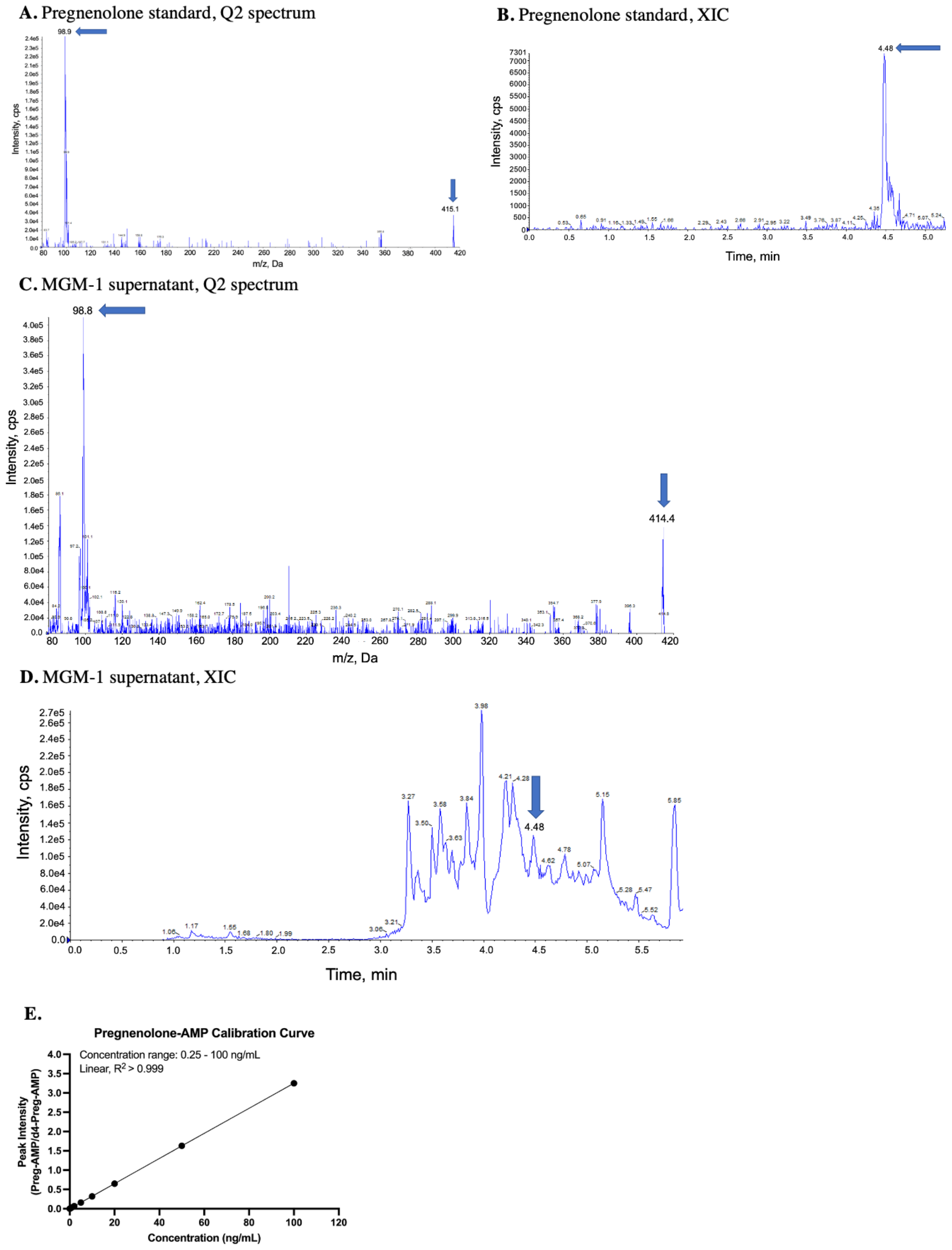
A. Pregnenolone standard, Q2 spectrum
98.9
415.1
Intensity, cps
m/z, Da
B. Pregnenolone standard, XIC
4.48
Intensity, cps
Time, min
C. MGM-1 supernatant, Q2 spectrum
98.8
414.4
Intensity, cps
m/z, Da
D. MGM-1 supernatant, XIC
3.98
4.48
Intensity, cps
Time, min
E.
Pregnenolone-AMP Calibration Curve
Concentration range: 0.25 - 100 ng/mL
Linear, R2 > 0.999
Peak Intensity (Preg-AMP/d4-Preg-AMP)
Concentration (ng/mL)

Figure S4: Mass spectrometry detection and quantification of pregnenolone production by MGM-1 and H295R-S1 cells. (A, C) Product ion scan for pregnenolone-AMP in derivatized samples of 200 ng/mL pregnenolone standard (A) and MGM-1 supernatant extract (C). (B, D) Extracted ion chromatograms (XIC) for pregnenolone-AMP (414.90 Da) in 1 ng/mL pregnenolone standard (B) and MGM-1 supernatant extract (D). (E) Calibration curve of pregnenolone-AMP. Under basal conditions, normalized pregnenolone-AMP concentration is 14.87 ng/mg protein in H295R-S1 supernatant and 0.32 ng/mg protein in MGM-1 supernatant, calculated based on the calibration curve.

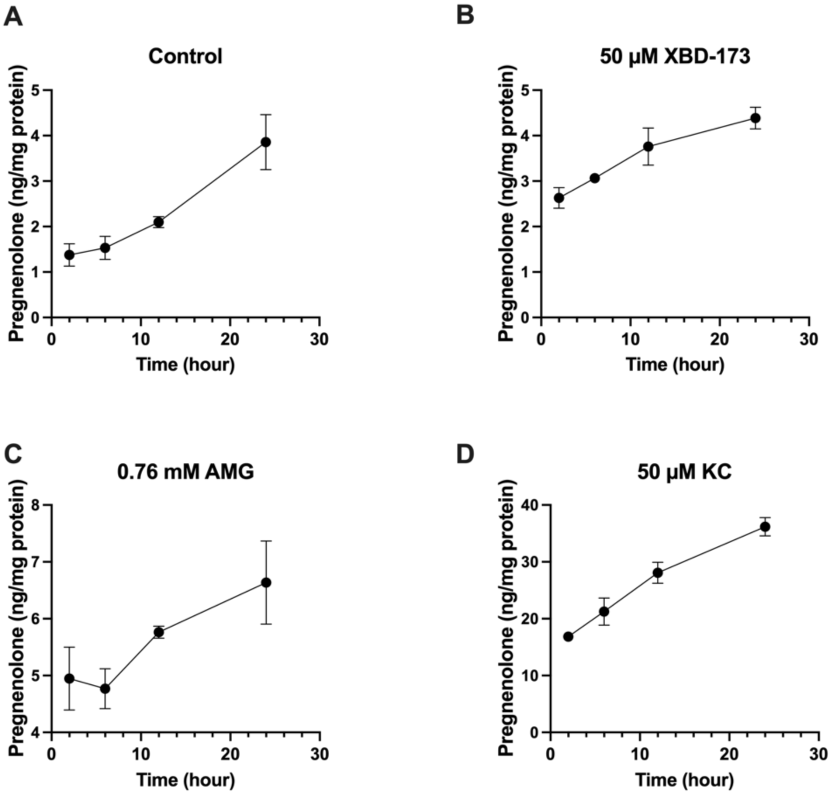

Figure S5: Pregnenolone secretion by MGM-1 cells over time. Measurement of pregnenolone in MGM-1 cell supernatants after 2, 6, 12, and 24 hours at basal levels (A) or with treatment of 50 µM XBD-173 (B), 0.76 mM AMG (C), or 50 µM ketoconazole (D). An accumulation of pregnenolone over time was observed in all conditions, indicating new synthesis and secretion of pregnenolone.

A. H295R-S1 supernatant steroid levels

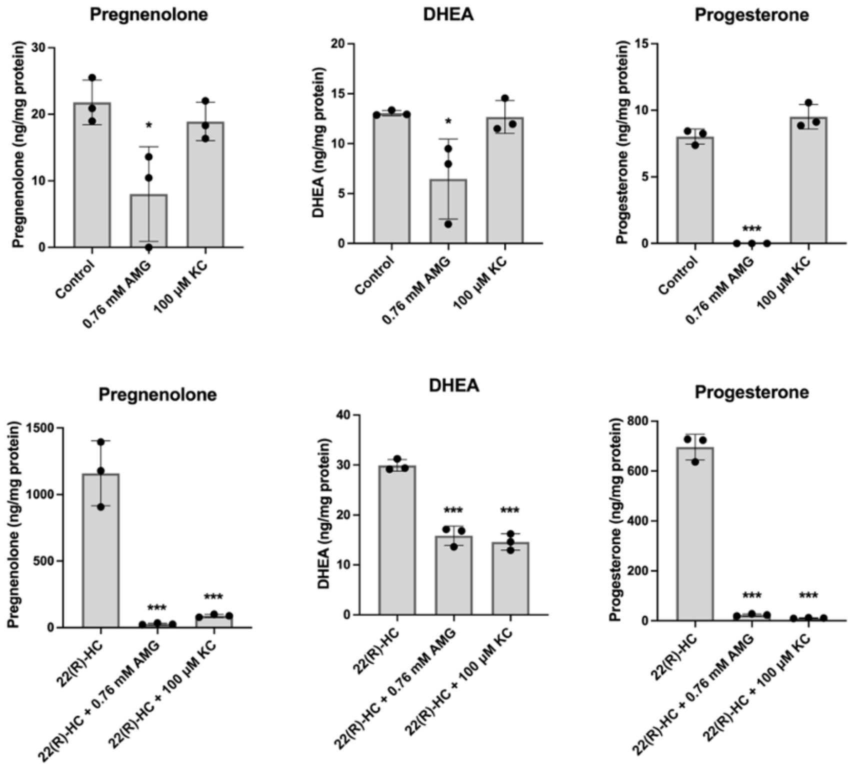

B. MGM-1 supernatant steroid levels

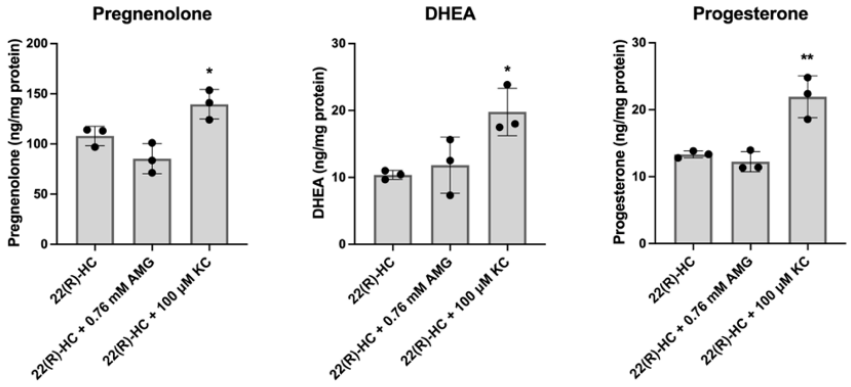

Figure S6: Steroid measurements of H295R-S1 and MGM-1 supernatants by mass spectrometry. Measurement of pregnenolone, DHEA, and progesterone in H295R-S1 (A) and MGM-1 (B) supernatants with or without 50 μM 22(R)-hydroxycholesterol stimulation and/or treatment with 0.76 mM AMG or 100 μM KC. Trends were similar to those observed using ELISA, where AMG and KC significantly inhibited steroid production in H295R-S1 cells but not MGM-1 cells. Pregnenolone levels produced by MGM-1 cells at basal levels (i.e., without hydroxycholesterol stimulation) were too low to detect using this mass spectrometry method. (* $p < 0.05$, ** $p < 0.01$, *** $p < 0.001$)

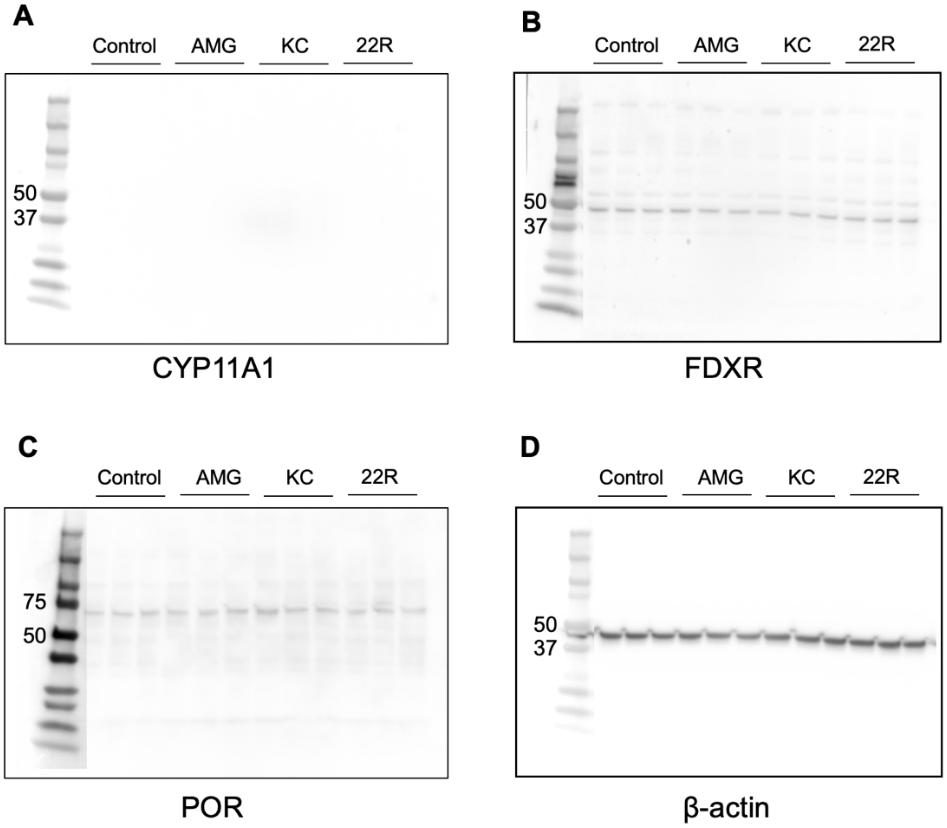

Figure S7: Western blot analyses of CYP11A1, FDXR, and POR in MGM-1 cells after treatment with high dose AMG, high dose KC or 22(R)-HC. Full membrane images of immunoblots shown in Figure 7D.

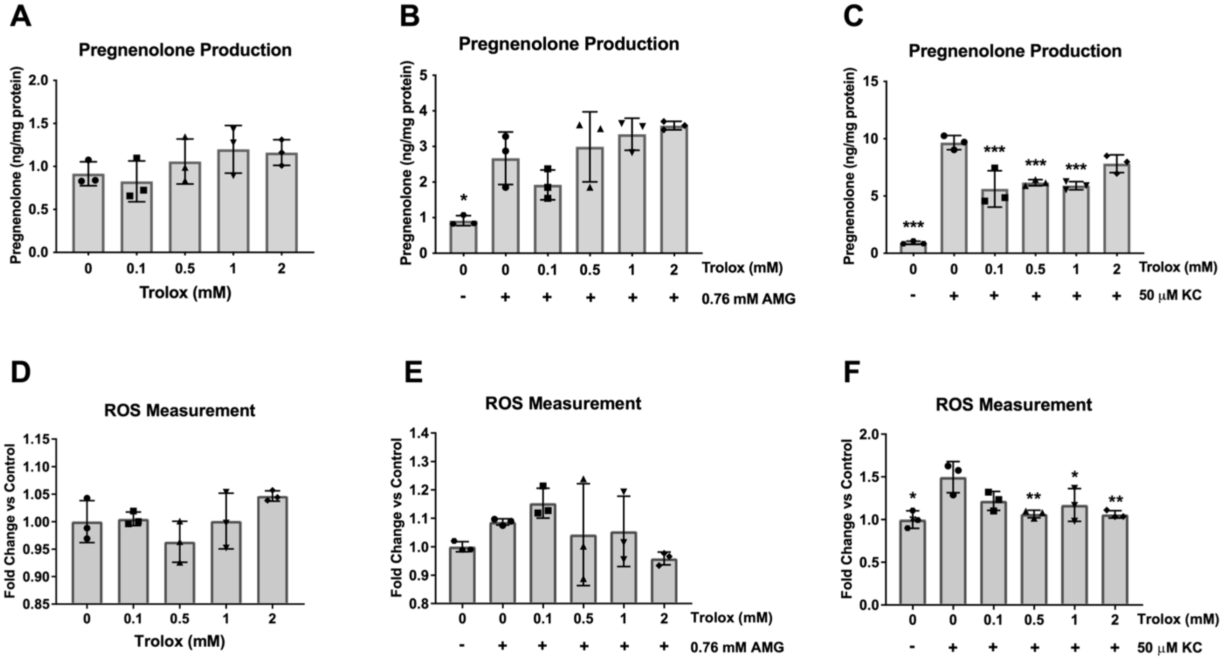

Figure S8: Effect of antioxidant Trolox on pregnenolone secretion and intracellular ROS in MGM-3 cells. ELISA measurements (A-C) of secreted pregnenolone and intracellular ROS measurements (D-F) when MGM-3 cells were treated with different doses of Trolox for 2 hours, either alone (A, D) or combined with 0.76 mM AMG (B,E) or 50 μM KC (C, F). Each data point represents the average of one experiment, where each treatment was performed in triplicate within each experiment. Data are presented as mean ± SD, N=3. Statistics performed compared to 0 mM Trolox (A, D), 0 mM Trolox + 0.76mM AMG (B, E), or 0 mM Trolox + 50 μM KC (C, F). Trolox treatment alone did not significantly change secreted pregnenolone or ROS at any concentration. Although 0.76 mM AMG and 50 μM KC significantly increased pregnenolone production in MGM-3 cells, only 50 μM KC significantly increased cellular ROS. Low doses of Trolox decreased pregnenolone production when MGM-3 cells were treated with 50 μM KC, but this effect was not seen at the highest dose 2 mM Trolox. Higher doses of Trolox decreased the elevation of cellular ROS induced by 50 μM KC. However, the trends in pregnenolone production and ROS when MGM-3 cells were treated with 50 μM KC combined with Trolox do not correlate. (* $p < 0.05$, ** $p < 0.01$, *** $p < 0.001$)

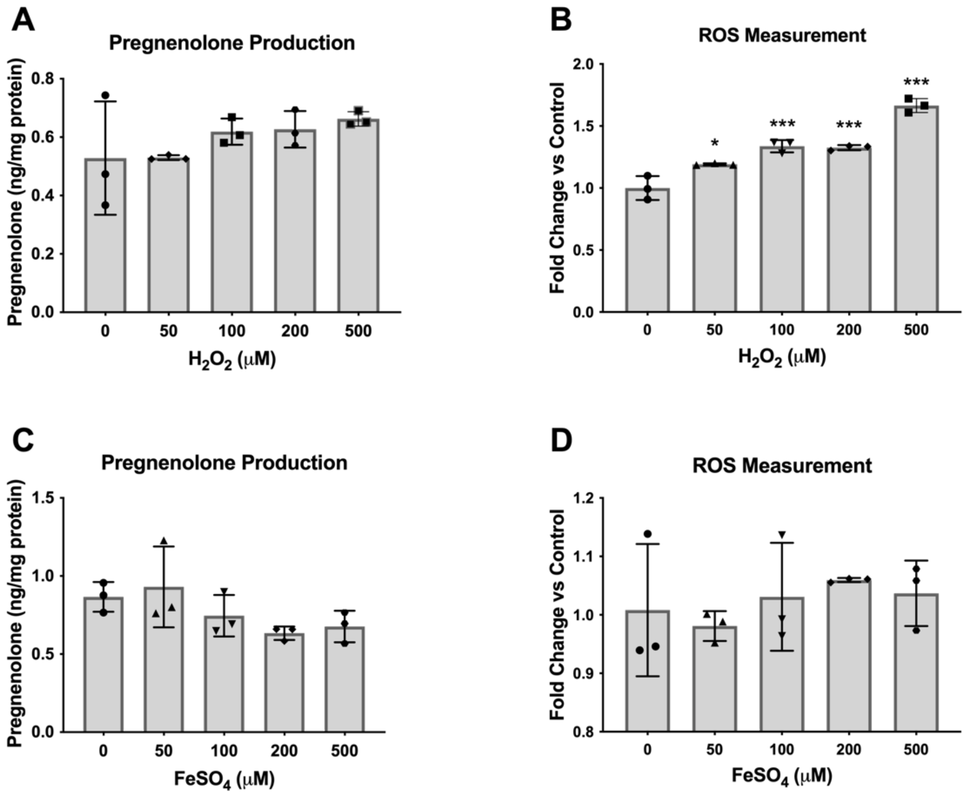

Figure S9: Effect of oxidants on pregnenolone secretion and intracellular ROS in MGM-3 cells. ELISA measurements (A, C) of secreted pregnenolone and intracellular ROS measurements (B, D) when MGM-1 cells were treated with different doses of hydrogen peroxide (H_2O_2) (A, B) or ferrous sulfate ($FeSO_4$) (C, D) for 2 hours. Each data point represents the average of one experiment, where each treatment was performed in triplicate within each experiment. Data are presented as mean ± SD, N=3. Statistics performed compared to the no treatment group within each panel. Hydrogen peroxide treatment significantly increased intracellular ROS starting at 100 μM but did not alter pregnenolone secretion by MGM-1 cells. Ferrous sulfate treatment did not significantly increase intracellular ROS except at 500 μM and did not change pregnenolone production by MGM-1 cells. (* $p < 0.05$, *** $p < 0.001$)

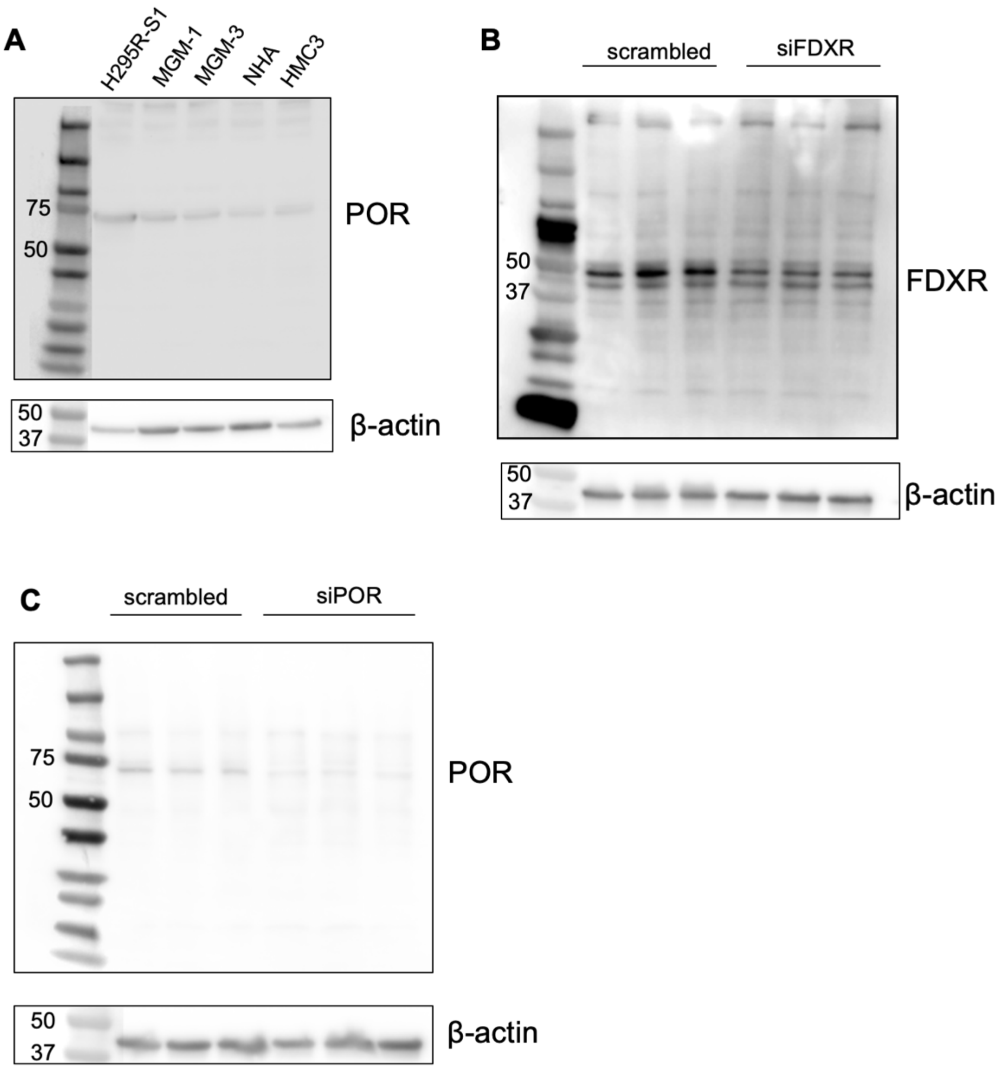

Figure S10: Western blot analysis of POR in human cell lines and confirmation of siRNA knockdown in MGM-1 cells. Full membrane images of immunoblots shown in Figure 14B, 15B, and 15F.
